# An alcove at the acetyl-CoA synthase nickel active site is required for productive substrate CO binding and anaerobic carbon fixation

**DOI:** 10.1016/j.jbc.2024.107503

**Published:** 2024-06-27

**Authors:** Seth Wiley, Claire Griffith, Peter Eckert, Alexander P. Mueller, Robert Nogle, Séan D. Simpson, Michael Köpke, Mehmet Can, Ritimukta Sarangi, Kevin Kubarych, Stephen W. Ragsdale

**Affiliations:** 1 Department of Biological Chemistry, University of Michigan, Ann Arbor, Michigan, USA; 2 Department of Chemistry, University of Michigan, Ann Arbor, Michigan, USA; 3 LanzaTech Inc, Skokie, Illinois, USA; 4 Stanford Synchrotron Radiation Lightsource, SLAC National Accelerator Laboratory, Menlo Park, California, USA

**Keywords:** carbon dioxide, carbon fixation, carbon monoxide, metalloenzyme, nickel, iron-sulfur protein, gas channel, one-carbon metabolism, bacterial metabolism

## Abstract

One of the seven natural CO_2_ fixation pathways, the anaerobic Wood-Ljungdahl pathway (WLP) is unique in generating CO as a metabolic intermediate, operating through organometallic intermediates, and in conserving (*versus* utilizing) net ATP. The key enzyme in the WLP is acetyl-CoA synthase (ACS), which uses an active site [2Ni-4Fe-4S] cluster (A-cluster), a CO tunnel, and an organometallic (Ni-CO, Ni-methyl, and Ni-acetyl) reaction sequence to generate acetyl-CoA. Here, we reveal that an alcove, which interfaces the tunnel and the A-cluster, is essential for CO_2_ fixation and autotrophic growth by the WLP. *In vitro* spectroscopy, kinetics, binding, and *in vivo* growth experiments reveal that a Phe229A substitution at one wall of the alcove decreases CO affinity thirty-fold and abolishes autotrophic growth; however, a F229W substitution enhances CO binding 80-fold. Our results indicate that the structure of the alcove is exquisitely tuned to concentrate CO near the A-cluster; protect ACS from CO loss during catalysis, provide a haven for inhibitory CO, and stabilize the tetrahedral coordination at the Ni_p_ site where CO binds. The directing, concentrating, and protective effects of the alcove explain the inability of F209A to grow autotrophically. The alcove also could help explain current controversies over whether ACS binds CO and methyl through a random or ordered mechanism. Our work redefines what we historically refer to as the metallocenter "active site". The alcove is so crucial for enzymatic function that we propose it is *part of* the active site. The community should now look for such alcoves in all "gas handling" metalloenzymes.

An existential threat facing humanity is the increasing global concentration of carbon dioxide in the atmosphere, affecting wide-reaching aspects such as rising sea levels, ocean acidification, and severe climate conditions (1, 2). There is an urgent need to better understand carbon fixation and apply this information to enhance CO_2_ uptake from the atmosphere and efficiently convert it into fuels and valuable chemicals (3, 4, 5). There are seven known CO_2_ fixation pathways and among these, the Wood-Ljungdahl (or reductive acetyl-CoA) pathway (WLP) exhibits high energy efficiency and is the only one that generates net ATP (Fig. 1*A*). The WLP is widely distributed in nature and is responsible for the production of acetic acid, an important chemical intermediate and microbial nutrient in anaerobic environments, including the gastrointestinal tracts of humans and ruminants, soil, bogs, marshes, and sediments (6). Found in strictly anaerobic microbes, this pathway allows acetogenic microbes to grow on H_2_ and CO_2_ as their sole energy and carbon sources and is important to the origin and evolution of life, as it was present in the last universal common ancestor (7). The WLP can also drive growth of anaerobic methanotrophic archaea on methane (8) and operate in reverse to allow microbes, *e.g.*, methanogens and sulfate reducers, to grow on acetate (9). The WLP is unique among biochemical pathways in using both CO_2_ and CO, generating CO as a metabolic intermediate, operating through organometallic intermediates, and generating acetyl-CoA as an energy source and metabolic building block (9) (Fig. 1*A*). Here, we report that a molecular alcove in the key WLP enzyme, acetyl-CoA synthase (ACS), is required for CO_2_ fixation. Without this alcove, the WLP cannot function because ACS cannot productively bind CO to form the key Ni-CO intermediate.

**Figure 1:**
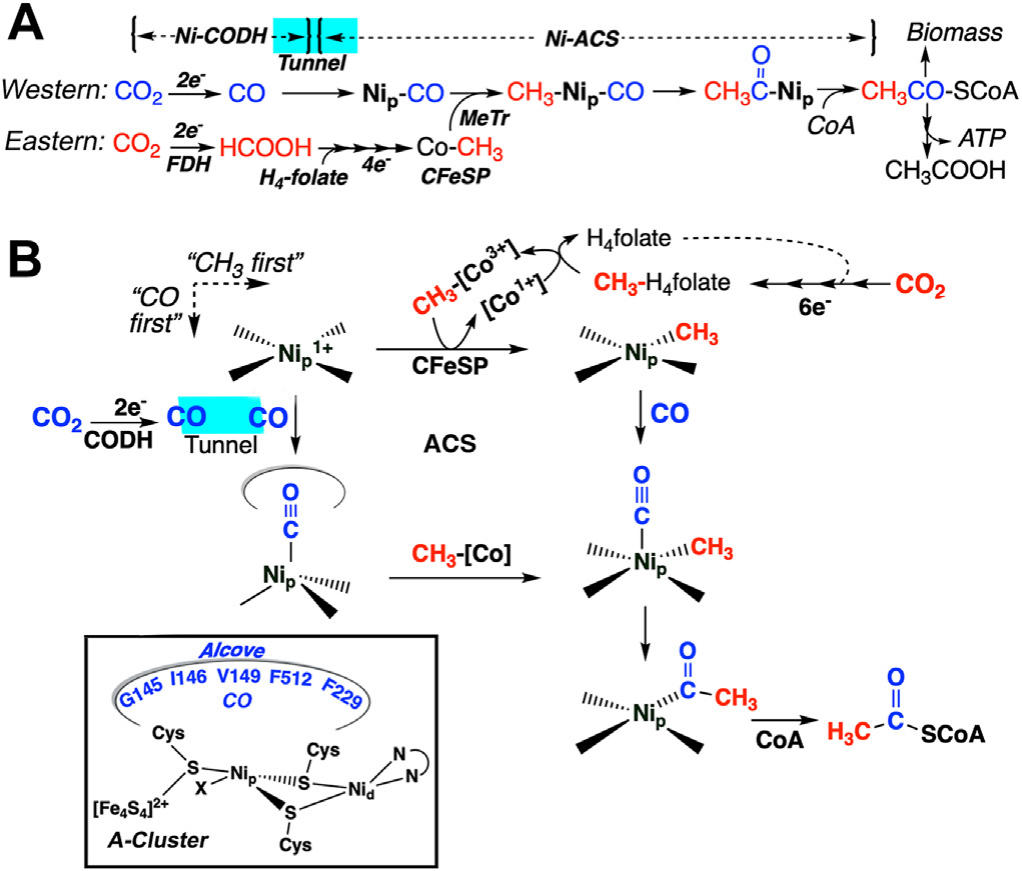
**An alcove in the Wood-Ljungdahl pathway (WLP) of anaerobic CO**_**2**_**fixation.***A*, focus on CODH, ACS, and the organometallic nature of the pathway. One CO_2_ enters the WLP at the Eastern (Methyl) branch (*red*), where it undergoes 2-electron reduction to formate by FDH), followed by 4-electron reduction to a methyl group methyltetrahydrofolate (CH_3_-THF), and transferred to the B_12_ center of the CFeSP (Co-CH_3_). In the Western (carbonyl) branch (*blue*), the other CO_2_ undergoes enzymatic 2-electron reduction by CODH to CO, which migrates to the Ni_p_ site in the A-cluster of ACS through a tunnel, which is tightly interfaced to the A-cluster. The A-cluster reacts with CH_3_-[Co] to generate CH_3_-Ni, binds CO, and undergoes C-C bond formation to form acetyl-Ni, and then reacts with CoA to generate acetyl-CoA, which serves as a metabolic building block for all carbon-containing molecules in the organism and as a source of ATP, through the enzymatic reactions of phosphotransacetylase and acetate kinase. *B*, catalytic mechanism of ACS and introduction of the alcove in the WLP. The Ni_p_ site of the A-cluster of ACS react first with either CO or methyl-Co, generated as described above, then the other substrate, and finally with CoA. The A-cluster and the residues of the Alcove are depicted in the inset. See the text for further details. ACS, acetyl-CoA synthase; CODH, carbon monoxide dehydrogenase; CFeSP, the corrinoid iron-sulfur protein; FDH, formate dehydrogenase; WLP, Wood-Ljungdahl pathway.

The central enzymes in the WLP are carbon monoxide dehydrogenase (CODH) and ACS, encoded by the *acsA* and *acsB* genes, which assemble into a heterotetrameric (αβ)_2_ enzyme complex (CODH/ACS) containing seven [Fe_4_S_4_] clusters (9) (Fig. 2). Each αβ dimer, contains two unique nickel clusters: the C-cluster of CODH (a Ni-Fe_4_S_4_ cluster), which catalyzes CO_2_ reduction to CO, and the A-cluster of ACS (a Ni_2_-Fe_4_S_4_ cluster (Figs. 1*B* and 2), which is responsible for acetyl-CoA synthesis. CO is generated at the CODH C-cluster and then migrates 70 Å through an interprotein gas tunnel to the ACS A-Cluster (10), where one (Ni_p_) of the two Ni A-cluster binds nascent CO and a methyl group, donated by the methylated corrinoid iron-sulfur protein (CFeSP), to form Ni-CO (11, 12) or Ni-methyl intermediates (13) (Fig. 1*A*). ACS can productively and randomly bind either CO or methyl as the first substrate (14), then catalyze C-C bond formation to form a Ni-acetyl (13) intermediate, which reacts with CoA to generate acetyl-CoA (Fig. 1*B*) (9).

**Figure 2:**
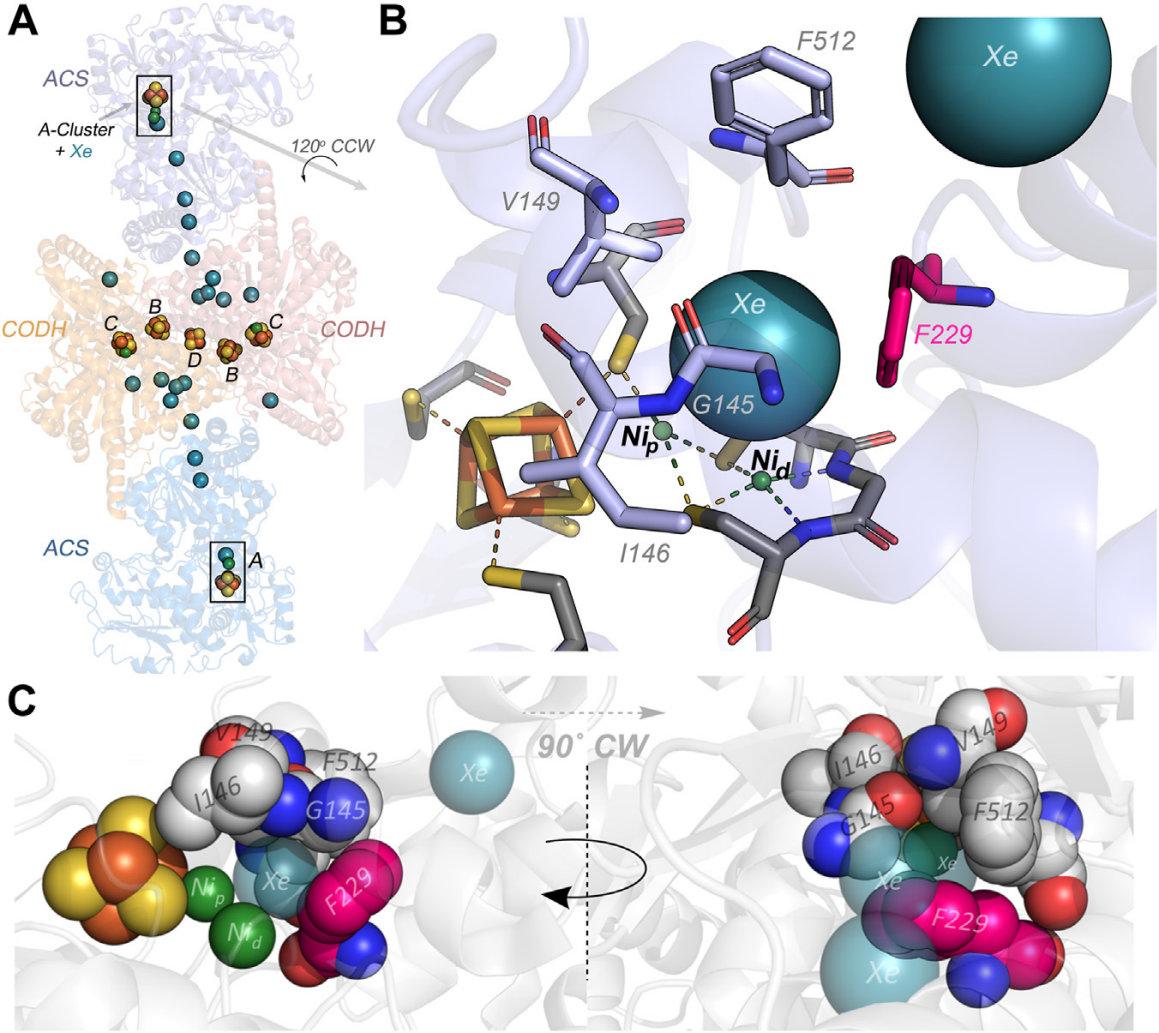
**Crystal structure of Xe-bound CODH-ACS, with focus on the alcove in ACS.** Crystal structure of (*A*) the CODH/ACS complex obtained under high-pressures of Xenon (*teal spheres*) to reveal the gas channel and show the 7 clusters in CODH (B–D) and ACS (A) involved in electron transfer and catalysis (C and A). *B*, expanded view of the A-cluster of ACS, highlighting the CO tunnel spanning from the CODH C-cluster (not shown) to the ACS A-cluster, with nearby residues as *spheres*. The residues that form the Alcove (Fig. 1*B*) are shown surrounding one of the Xe atoms. *C*, side-on perspective of the A-cluster, showing the alcove’s role in maintaining CO near Ni_p_, with F229 shown in *magenta*. (*left*) Down-tunnel perspective, 90˚ CW toward viewer (relative to (*right*)), showing how F229 and nearby residues form an opening for CO to reach the A-cluster. Stereoscopic versions of these images are found in the supplemental materials (Figs. S4 and S5). PDB 2Z8Y. ACS, acetyl-CoA synthase; CODH, carbon monoxide dehydrogenase; CW, clockwise; PDB, Protein Data Bank.

Earlier kinetic, spectroscopic, and structural studies identified a constellation of hydrophobic residues at the end of this tunnel near the proximal nickel center in the A-cluster (10, 15, 16) (Figs. 1*B* and 2). The X-ray crystallographic structure of CODH/ACS, obtained under a xenon overpressure, revealed a xenon atom cradled in a hydrophobic basket of residues (F229, I146, F512, V149, and G145) at the end of the CO tunnel only 3.5 Å from Ni_p_ of the A-cluster (Fig. 2) (10). Bender, *et al.* (15) showed that low-temperature photolysis of the Ni-CO intermediate generates a species that lacks the characteristic Ni(I)-C≡O electron paramagnetic resonance (EPR) and infrared (IR) signals and yields a new EPR signal corresponding to a CO-cleaved Ni_p_(I) species. Temperature-dependent kinetic studies revealed an activation energy barrier for CO rebinding of <1 kJ/mol, suggesting that CO migrates freely between Ni_p_ and a putative “alcove”. Here, we use *in vitro* and *in vivo* experiments to compare the properties of variant enzymes in two acetogens (*Moorella thermoacetica* and *Clostridium autoethanogenum*) with the WT proteins and establish that this CO alcove is required for productive interactions between ACS and substrate CO to form the key Ni-CO intermediate in the WLP. Disruption of this alcove prevents CO_2_ fixation and autotrophic growth by this pathway. This work demonstrates that the CO alcove is a required and integral component of the A-cluster active site.

## Results

### An intact alcove is required for productive carbonylation of ACS

To investigate the role of the alcove, we made substitutions in a residue (F229) that forms one of its walls in the well-characterized ACS from *M*. *thermoacetica* and compared the properties of F229A and a conservative F229W with those of WT ACS (Table 1). We hypothesized that this mutation would open one wall of the alcove by replacing the bulky benzene group of Phe (accessible surface area, 220.0 A^2^) (17) with the small hydrophobic methyl group in Ala (121.0 A^2^), and examined the effect of substitution with Trp (264.0 A^2^). Both variants exhibit a metal content very similar to that of WT protein, consistent with near complete occupancy of the A-cluster (Table S1).

**Table 1:** Monitoring methylation activity of ACS variants by single-turnover stopped-flow kinetics

Variant	390 nm			470 nm		
	k_obs_ (s^−1^)^a^	Relaxation factor or stretch (*β*)^a^	Max conversion percent^b^	k_obs_ (s^−1^)^a^	Relaxation factor or stretch (*β*)^a^	Max conversion percent^b^
WT	0.0464 (±0.0006)	0.575 (±0.005)	84.5 (±0.2)	0.0781 (±0.0009)	0.783 (±0.009)	57.8 (±0.6)
F229W	0.180 (±0.002)	0.573 (±0.004)	71 (±1)	0.164 (±0.002)	0.709 (±0.008)	60 (±2)
F229A	0.124 (±0.001)	0.533 (±0.005)	74.4 (±0.6)	0.105 (±0.002)	0.72 (±0.01)	74 (±2)

a The methylation data (Fig. 7) are best fit by a stretched exponential, which includes a “relaxation” factor (β) allowing for a distribution of rate constants. The equation for the stretched exponential fit is y = c + Ae^(−(kt)ˆβ)^, where 0 < β ≤ 1.

b Maximum percent conversion of MeCbi to Co(I)-Cbi based on their extinction coefficients. At 390 nm, ε_390,MeCbi_ = 8 mM^−1^ cm^−1^ and ε_390,Co(I)-Cbi_ = 25 mM^−1^ cm^−1^, so Δε_390_ = 17 mM^−1^. At 470 nm, ε_470,MeCbi_ = 11 mM^−1^ cm^−1^ and ε_470,Co(I)bi_ = 3 mM^−1^ cm^−1^, thus Δε_470_ = 8 mM^−1^ cm^−1^.

**Figure 3:**
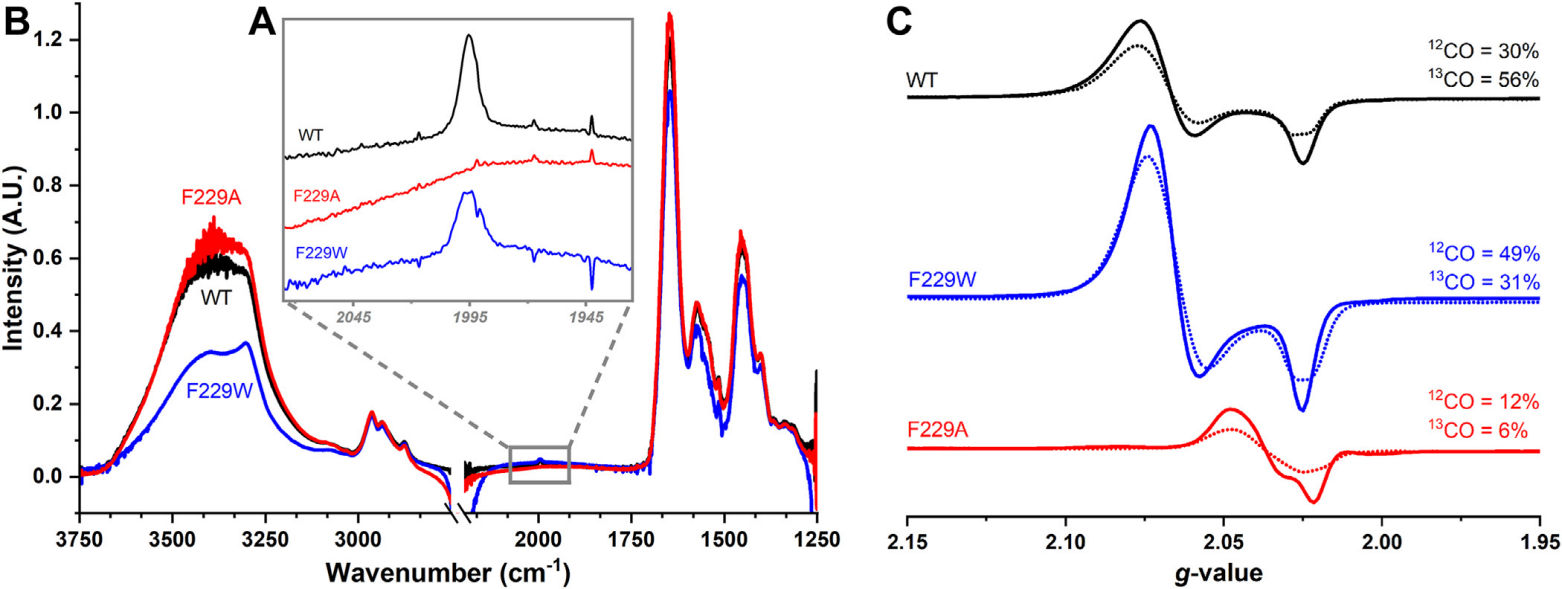
**FTIR and EPR spectra of ACS variants.***A*, FTIR: highlight of the characteristic Ni-CO IR stretch at 1995 cm^−1^ between the region 1970 to 2020 cm^−1^. The Ni-CO stretch is noticeably absent in F229A, while the WT and F229W have strong Ni-CO signals. The integrated area of the F229W Ni-CO peak is 83% of the WT area. The protein concentrations were as follows: WT, 580 ± 12 μM; F229W, 590 ± 26 μM, and F229A, 580 ± 16 μM. *B*, the full spectra of the ACS variants showing global folding similarity. Bands belonging to ACS include the various amide bands over the 1250 to 1750 cm^−1^, the C-H bonds from 2800 to 3000 cm^−1^, and the N-H and O-H stretches from 3200 to 3750 cm^−1^. The region between 2200 to 2750 cm^−1^ was omitted due to strong D_2_O signals obscuring the spectra. *C*, EPR spectra of the ACS variants exposed to naturally abundant ^12^CO (––) compared with isotopically labeled ^13^CO (---). Quantified spin percentages are listed on the right as NA%/^13^CO%. The ^13^C splitting is most prominent at *g* = 2.028. Less intense broadening can be seen at 2.08 for WT and F229W, and at 2.062, 2.057, and 2.024 for the F229A variant. The protein concentrations for the EPR were 236 μM (WT), 280 μM (F229A), 276 μM (F229W) and the spin concentrations are shown. ACS, acetyl-CoA synthase; EPR, electron paramagnetic resonance; IR, infrared; NA, natural abundance.

CO binding to ACS was directly monitored by FTIR and EPR spectroscopies. Incubation of the F229W variant with CO leads to an intense 1995 cm^−1^ FTIR signal, which is assigned to the stretching frequency of the nickel-carbonyl of the A-cluster (18) and is similar to that of the WT protein (Fig. 3*A*). Transient kinetic experiments have shown that this Ni(I)-CO IR band appears upon reaction of CODH/ACS with CO (19) and disappears at catalytically competent rates (*i.e.* faster than the steady-state rate of acetyl-CoA synthesis) when the Ni-CO complex is reacted with the methylated CFeSP (20).

While the F229W variant was proficient at CO binding, the F229A variant lacks any obvious FTIR signal in the 1980 to 2150 cm^−1^ range (Figs. 3*A* and S1). However, the similarity of the FTIR spectra for the variants and WT proteins in the amide (1250–1750 cm^−1^) and C-H (2800–3000 cm^−1^) regions (Fig. 3) indicate that the mutations did not cause a broad protein disruption and that the decreased CO affinity of the F229A variant is not due to global protein misfolding.

When the ACS F229W variant was incubated with CO, formation of the paramagnetic Ni(I)-CO (NiFeC) species was observed by EPR (Fig. 3*C*). Its intensity and *g*-values (at 2.074 and 2.028) were characteristic of the WT protein (21). In contrast, the CO-incubated F229A variant showed 3- to 4-fold less intensity than that of WT and F229W and a marked shift in the *g*-values to 2.062, 2.046, and 2.028. By incubating ACS with ^13^CO, line broadening of the EPR spectrum was observed for all variants, unambiguously demonstrating that CO binds to their A-clusters (21). The EPR spectrum of F229A resembles that of a minority species sometimes observed in WT ACS (22) and of the CO-treated methanogenic ACS (23), which lacks the first 320 amino acids (including F229) of the bacterial enzyme. Recent electron nuclear double resonance (ENDOR) results reveal that the low intensity EPR spectrum of F229A consists of three components including one assigned to a Ni(I) species that lacks CO (16). The NiFeC signal of F229A, like those of WT and F229W, forms during mixing (ca. 30 s) and does not increase with incubation time for up to 1 h.

**Figure 4:**
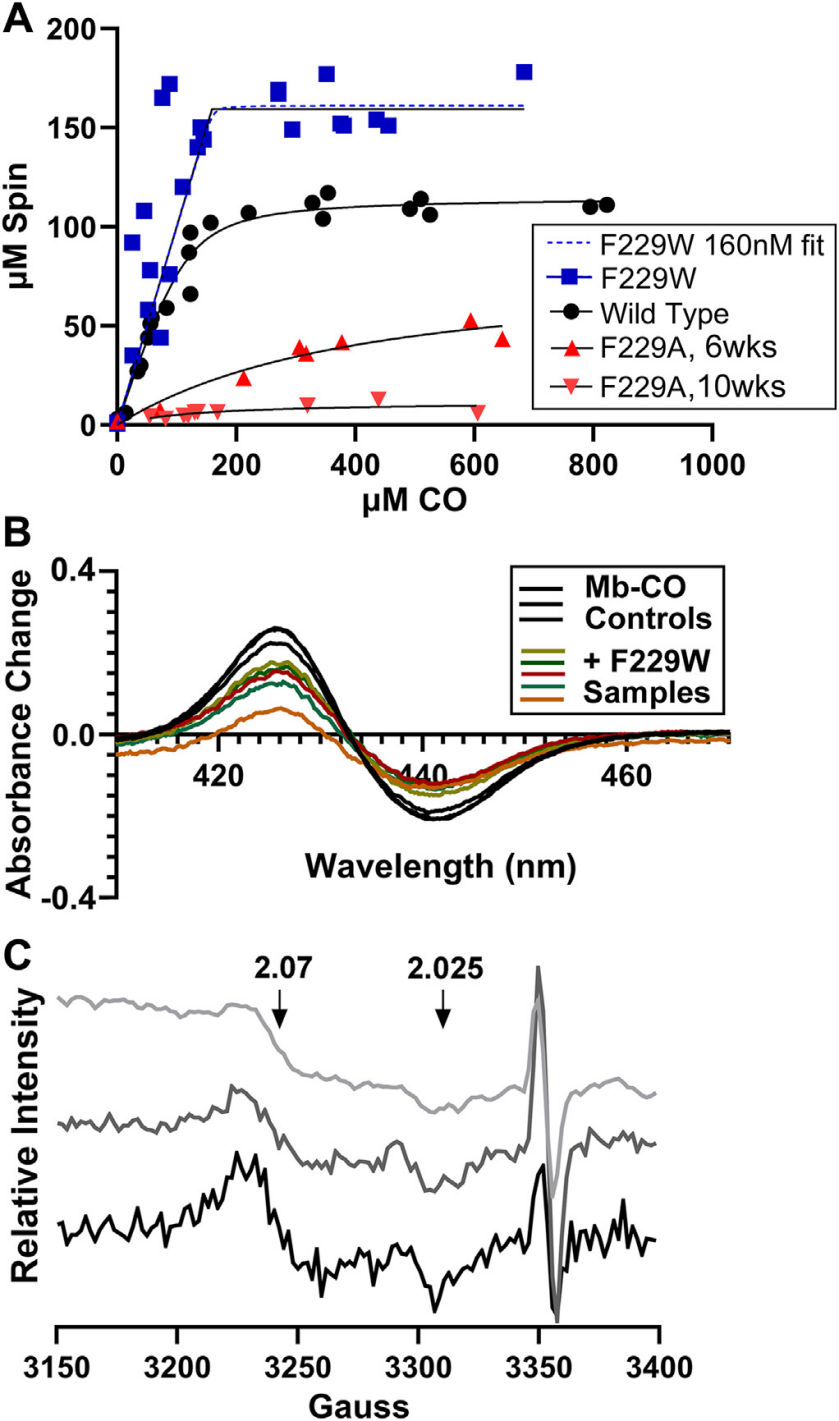
**Comparison of the CO binding affinity of the alcove variants.***A*, direct CO titration showing individual data points at varying CO concentrations for the ACS variants fit to a quadratic tight-binding equation (see Experimental procedures). EPR samples of ACS were exposed to increased concentration of CO (concentration measured by binding at 424 nm to equine Mb), frozen in liquid N_2_, and placed in an EPR cryostat maintained at 100 K for EPR spectroscopy. The spectrum was doubly integrated and spin quantified by comparison to a Cu(II) perchlorate standard. The measured *K*_*D*_ values (and the 95% confidence intervals) were 12.8 μM (7.3–20.2 μM, 95% CI), 0.16 μM (<1 pM-8 μM, 95% CI), and 340 μM (140–940 μM at 95% CI), respectively, for WT, F229W, and F229A. *B* and *C*, anaerobic equilibrium CO competition experiment between F229W and dithionite-reduced (Fe^2+^) equine myoglobin (Mb). In (*B*), UV-visible spectra of triplicate Mb-CO control samples were determined from a solution of 4 μM Fe^2+^-Mb reacted with 4.0 μM CO in 50 mM Tris–HCl, pH 7.6 in a 1 cm cuvette at 25 ^°^C. For the quintuplicate competition experiments, 4.0 μM CO was added to 4 μM active F229W, sealed with a rubber septum, equilibrated for 5 min, and an initial spectrum was recorded. Then, 4 μM Fe^2+^-Mb was added, incubated for 30 min, and the equilibrium spectrum was recorded. The difference spectra (Fe^2+^-Mb-CO minus Fe^2+^-Mb) are shown. Based on the five UV-visible experiments, the K_D_(CO) for F229W was 160 ± 30 nM (95% confidence interval, CI). In (*C*), triplicate EPR experiments were performed in which 200 μl samples were transferred from the UV-visible cuvette to a quartz capillary, quickly frozen in liquid N2, and EPR spectra were run to determine the amount of F229W-CO. Based on the EPR measurements, the average K_D_ value was 160 ± 90 nM (95% CI). Thus, the three K_D_(CO) measurements (from the direct CO binding and CO competition experiments) reveal a K_D_(CO) for the F229W-CO complex between 150 to 190 nM at a 95% confidence level. ACS, acetyl-CoA synthase; CI, confidence interval; EPR, electron paramagnetic resonance.

CO-binding proficiency of the variants was measured by following the increase in intensity of their NiFeC EPR signals at increasing CO concentrations (Fig. 4). The F229A variant exhibits at least 30-fold lower (*K*_*D*_ = 340 μM) affinity for CO than WT ACS (*K*_*D*_ = 12.8 μM). At saturating CO levels, F229A also exhibits a NiFeC EPR signal that amounts to only 10 to 20% that of either the WT protein or of F229W.The value for the WT protein matches that measured earlier (23, 24, 25). On the other hand, F229W binds CO too tightly to accurately measure by this EPR-based approach (*K*_*D*_ << 1 μM), with a calculated *K*_*D*_ in the pM range, but an unreasonably wide confidence interval (CI).

**Figure 5:**
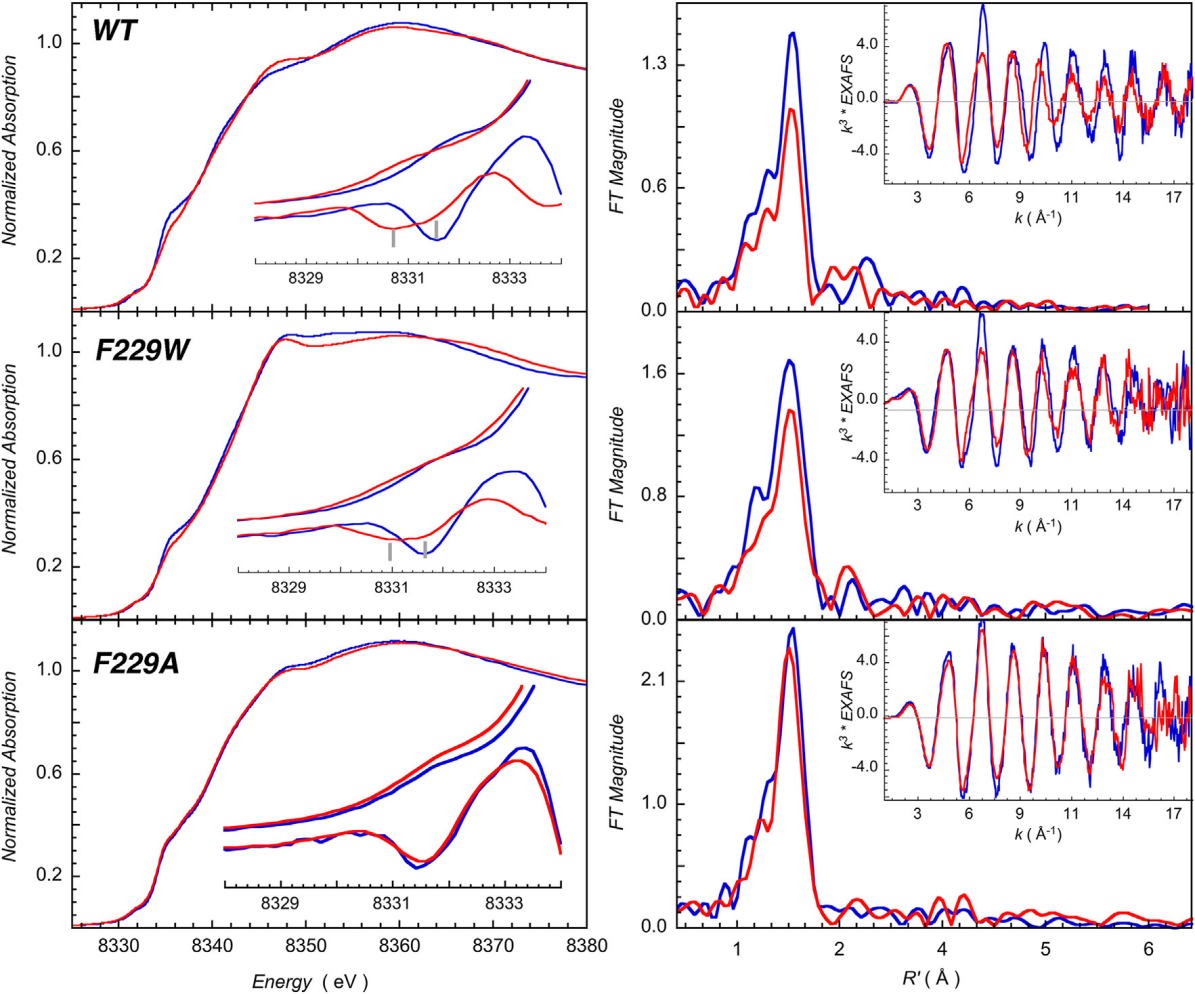
**Ni K-edge XAS data showing pre-edge changes on the left and related EXAFS on the right.***Insets* show shifts in the pre-edge for CO-exposed (*red*) ACS relative to unexposed (*blue*), where the first derivative shows distinct shifts for one of the two A-cluster Ni. Ni K-edge data for F229A does not show a change seen in the WT and F229W. Consistent with these pre-edge data are the EXAFS, which show little to no changes for CO-exposed F229A relative to unexposed. WT data adapted with permission from ref (11). Copyright 2017 American Chemical Society. ACS, Acetyl-CoA synthase; EXAFS, extended X-ray absorption fine structure; XAS, X-ray absorption spectroscopy.

Because the CI for the K_D_(CO) in the direct CO binding experiments (Fig. 4*A*) with F229W was so wide, we used an anaerobic equilibrium CO competition experiment between F229W and dithionite-reduced (Fe^2+^) equine myoglobin (Mb), which exhibits a K_D_(CO) of 22 nM (26). In this approach, we added Fe^2+^-Mb to F229A-CO in the anaerobic chamber, allowed them to reach equilibrium, and measured the initial and final equilibrium amounts of Mb-CO by UV-visible spectroscopy (Fig. 4*B*) and F229W-CO by EPR spectroscopy (Fig. 4*C*). The three K_D_(CO) measurements obtained from the direct CO binding and Mb-CO/F229W-CO competition approaches converge on a K_D_(CO) for the F229W-CO complex between 130 to 190 nM at a 95% confidence level. Thus, the Kd(CO) varies over 2125-fold (from 0.160 to 340 μM), depending on the identity of the alcove residue at position 229. To understand how mutation of a second-sphere aromatic residue near Ni_p_ could disrupt formation of the organometallic Ni_p_-CO bond, we performed Ni and Fe K-edge X-ray absorption spectroscopic (XAS) and high-*k* extended X-ray absorption fine structure (EXAFS) experiments for the three variants in the presence and absence of CO and compared the preedge, near, and extended regions of the Ni (Fig. 5) and Fe (Fig. S3) absorption edges. Ni- (or Fe-) XAS experiments detect all the Ni (or 4 Fe’s) in the sample; thus, this experiment reflects the average Ni_p_, Ni_d_, and Fe structural and electronic states. Before exposure to CO, each of the variants treated with dithionite display a nearly identical Ni K-preedge features at 8331.7 eV, indicating that their Ni_p_ (and Ni_d_) centers remain unchanged by the F229 substitutions. The differences at higher energies (∼8348 eV and higher) between WT and F229W may indicate changes in long-range multiple scattering effect due to the presence of the bulkier tryptophan ligand. Similarly, upon addition of CO to WT ACS or the F229W variant caused a similar decrease in the Ni K rising-edge intensity (indicating tetrahedral geometry at Ni_p_), splitting of the Ni K-preedge (electronic structure change at Ni_p_ upon CO binding) and damping of the EXAFS signal (suggesting elongation of the Ni_p_-S bond upon CO binding) as seen earlier for the WT protein (11) (Fig. 5). Thus, the F-to-W mutation has little to no effects on structure of Ni(I)-CO.

Fig. S3 shows the Fe K-edge XAS data for the WT, F229A, and F229W species (treated with dithionite) before and after exposure to CO. The data unambiguously show no change upon CO binding supporting the assignment of Ni_p_ as the sole CO binding site in the A-cluster, in accordance with prior XAS (11) and X-ray diffraction (12) studies as well as EPR and FTIR experiments triggered by photolysis (15) and stopped flow (19) instrumentation.

Thus, the similarity of the Ni- and Fe-XAS data for the dithionite-reduced WT and F229 variants, which demonstrate that the A-cluster is properly formed, support the methylation data and the FTIR spectral results (above) indicating that the mutations did not disrupt the overall structure of ACS.

However, for the F229A variant, we observed no significant differences in the Ni K-preedge, rising-edge or in the EXAFS region between the dithionite-reduced and CO-exposed protein (Fig. 5). Since CO-incubated F229A variant exhibits an EPR spectrum that shows ^13^CO splittings (Fig. 3*C*), the lack of changes in the Ni-preedge, XAS, or EXAFS on CO exposure likely reflects its four-fold lower CO binding proficiency (above), leaving most of the protein in its resting planar Ni_p_(II) state. Similarly, treatment of F229A-ACS with CO does not show differences in the Fe K-edge XAS data (Fig. S3). On the other hand, earlier ENDOR studies, which focus on the EPR-active components of the carbonylation reaction, demonstrated that the remaining paramagnetic Ni in the A-cluster of the F229A variant is composed of is a heterogeneous mixture of species, including a Ni_p_(I) species that lacks CO (16).

### An intact alcove is required for autotrophic growth by the WLP

**Figure 6:**
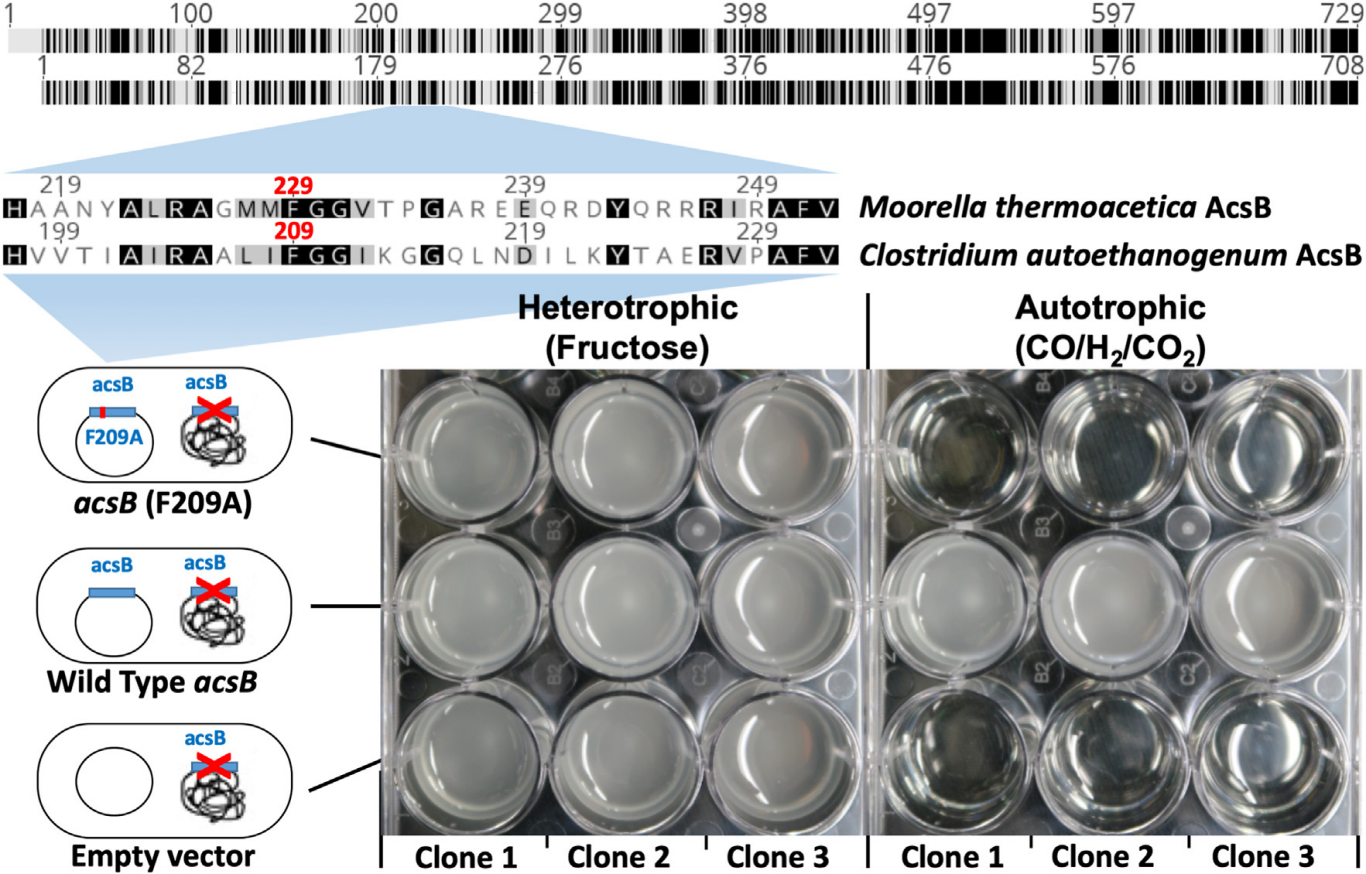
**(*****T******op*) Alignment of AcsB showing highly conserved region around AA position 229 in *Moorella thermoacetica* and corresponding position 209 in *Clostridium autoethanogenum* (*bottom*) Complementation of *C. autoethanogenum ΔacsB*.** Only wild-type *acsB* but not *acsB*(F209A) is able to restore growth under CO/H_2_/CO_2_ autotrophic conditions. ACS, Acetyl-CoA synthase; AcsB, bacterial ACS.

The kinetic and spectroscopic results described above suggest that the alcove may be essential for productive CO binding during anaerobic CO_2_ fixation. To test the effect of the alcove on growth by the WLP pathway, we chose *C*. *autoethanogenum* as the host, due to the robust genetic tools available for this acetogen (4) and previous demonstration on mutagenesis and plasmid based complementation of the CODH/ACS complex (27). Following the earlier study where *acsA* had been deleted, we applied the same strategy and successfully deleted the native ACS gene (*acsB)* in the genome of *C. autoethanogenum* and complemented the resulting *C. autoethanogenum* Δ*acsB* strain with both WT *acsB* and well as *acsB* (F209A). Although the *C. autoethanogenum* AcsB enzyme lacks the first 20 AA compared with the *M. thermoacetica* AcsB, the two proteins share 43% identity. The region around F229 of the *M. thermoacetica* enzyme is highly conserved among acetogens and corresponds to F209 of the *C. autoethanogenum* ACS (Fig. 6, top). The Δ*acsB* strains carrying the respective plasmids were tested, in biological triplicate, for their ability to grow heterotrophically on fructose and autotrophically on a blend of CO, CO_2_, and H_2_. All three strains were able to grow heterotrophically but, in the absence of WT *acsB*, strains strikingly did not grow autotrophically (Fig. 6, bottom). While the WT *acsB* could complement the genomic deletion in autotrophic growth, the F209A variant was unable to rescue the KO. Thus, *C*. *autoethanogenum* F209A *(M. thermoacetica* F229A) is unable to grow on syngas (CO/H_2_/CO_2_).

### The alcove is not required for methylation of ACS

The above results suggest that the alcove, which is critical for CO binding and for autotrophic growth by the WLP, plays a significant role in CO access, control, and precise binding to the ACS active site. Crystallographic and electron microscopic data (10, 12, 28, 29) demonstrate that the ACS-CO state exhibits a closed conformation that facilitates CO transport through the tunnel connecting the C-cluster of CODH to the A-cluster, with the alcove residues closing as a hand would grasp a ball and Ni_p_ adopting a tetrahedral Ni_p_(I)-CO coordination geometry (11, 12), as diagrammed in Figure 1*B*. However, methyl transfer from methyl-Co(III) to Ni_p_ requires an open conformation poised for binding the CFeSP and, in this state, the alcove residues (except F512) disperse (12, 13, 29). Thus, the transition between methylation and carbonylation would require alcove formation and dispersal at each catalytic cycle as a hand opens and closes to catch a ball. These major structural changes radiating out from the A-cluster provoke the question, is the alcove required for ACS methylation? Similarly, since the alcove seems to stabilize the open-channel/closed-conformation of ACS to facilitate CO binding, does the F229A substitution facilitate the open (extended) methylation-ready conformation?

**Figure 7:**
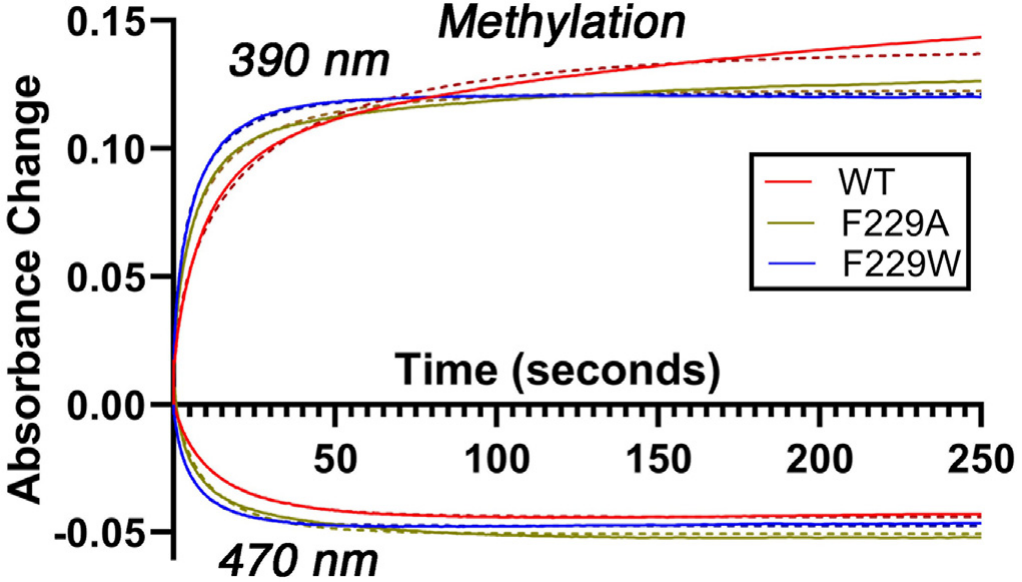
**Transient kinetics of methylation of ACS with MeCbi.** Stopped-flow traces of 10 µM ACS reduced with 3x Ti(III) citrate and methylated with 10 µM MeCbi measured by monitoring the time-dependent decrease in absorbance at 470 nm of MeCbi and the increase at 390 nm of Co(I)-Cbi. Data were fit with the equation for a stretched exponential. ACS, acetyl-CoA synthase; MeCbi, methyl-Cbi; Ti(III) citrate, titanium(III) citrate.

The methylation activity of the F229A variant was compared to those of WT and F229W by monitoring decrease in the UV-visible band at 470 nm, associated with methyl-cob(III)inamide (MeCbi) and increase in the Co(I)-cobinamide (Cbi) absorption peak at 390 nm (Figs. 7 and S2). We used MeCbi instead of the methylated CFeSP to allow rigorous characterization of the methyl-ACS product, because the CFeSP contains cobalt and an Fe_4_S_4_-cluster that would obscure any changes in the Fe_4_S_4_-cluster of ACS that might occur during methyl transfer. MeCbi has been shown to serve as an effective methyl donor to ACS, with a reaction mechanism identical to that with the methylated CFeSP, albeit 100-fold slower than the methylated CFeSP (30).

All three variants underwent methylation to similar extents (70%) and at similar rates (Table 1), indicating that the alcove is not required for methylation of ACS. These activity data also demonstrate that the F229 substitutions have not disrupted the A-cluster, in concurrence with the XAS, backbone FTIR, and metal determination results described above. They also conform with recent electron microscopic studies, which indicate that interactions between Ni_p_ and methyl-cob(III)inamide occur in an extended conformation (29) *versus* carbonylation which requires a closed state, with F229 and the other alcove residues clustered around Ni_p_, and the CO channel, alcove, and Ni_p_ forming a cohesive unit (Fig. S6).

## Discussion

Much effort has been invested to trap and characterize the proposed organometallic intermediates in the WLP (Fig. 1). Now, just as this effort seemed to have come to fruition with characterization of the Ni-CO, Ni-CH_3_, and Ni-acetyl intermediates (11, 12, 13), we reveal here that we must broaden our metal-centric view of the active site of ACS. Here, we rigorously and quantitatively demonstrate that the Ni_p_ center in the A-cluster that forms the organometallic bonds, has insufficient affinity to bind CO at the low micromolar levels required for enzymatic catalysis and autotrophic (H_2_/CO_2_) growth. The ACS active site requires augmentation by an alcove consisting of five highly conserved hydrophobic amino acid residues, which form an alcove connected directly to the A-cluster and the gas tunnel. A Xe-pressurized structure of CODH/ACS (10), provided a glance at the alcove. This structure revealed a xenon atom cradled in a hydrophobic basket of residues between Ni_p_ of the A-cluster on one side and the CO tunnel on the other (Fig. 2). A structure-function relationship and the term “alcove” were proposed in an article describing a very low barrier for substrate CO to rebind to Ni_p_ after photolytic dissociation (15). Here, we reveal that the alcove is required for productive binding of substrate CO during anaerobic carbon fixation by the WLP. *In vitro* and *in vivo* experiments reveal that substitution of the bulky benzene group of Phe229 at one wall by the small methyl group of Ala decreases CO affinity thirty-fold and abolishes autotrophic growth by the WLP. However, the F229A variant is otherwise competent for A-cluster assembly and reaction with the methyl donor. Since disrupting Ni-CO formation obstructs the WLP, is it possible that enhancing CO binding could improve the pathway? Replacement of the benzene group of F229 with the larger indole of Trp enhances CO affinity 80-fold without affecting methyltransferase activity. Our work redefines what we historically refer to as the metallocenter "active site". Thus, as serine proteases like carboxypeptidase lack biological function without an intact tripartite active site consisting of a catalytic triad (Ser195, Asp102, and His57), an electropositive oxyanion hole (the backbone NHs of Gly193 and Ser195), and a polypeptide substrate recognition/binding site (31), the A-cluster alone is not a productive catalyst and requires several key functions of the alcove for concentrating and directing CO to the metallocofactor and preventing CO loss and inhibition. As ACS contains a tunnel that, like in methane monooxygenase (32), serves as an extension of the active site, we propose that the alcove should be considered part of the ACS active site and suggest that the community seek similar alcoves in the active site of other gas-handling enzymes, *e.g.*, formate dehydrogenase, hydrogenase, and nitrogenase.

**Figure 8:**
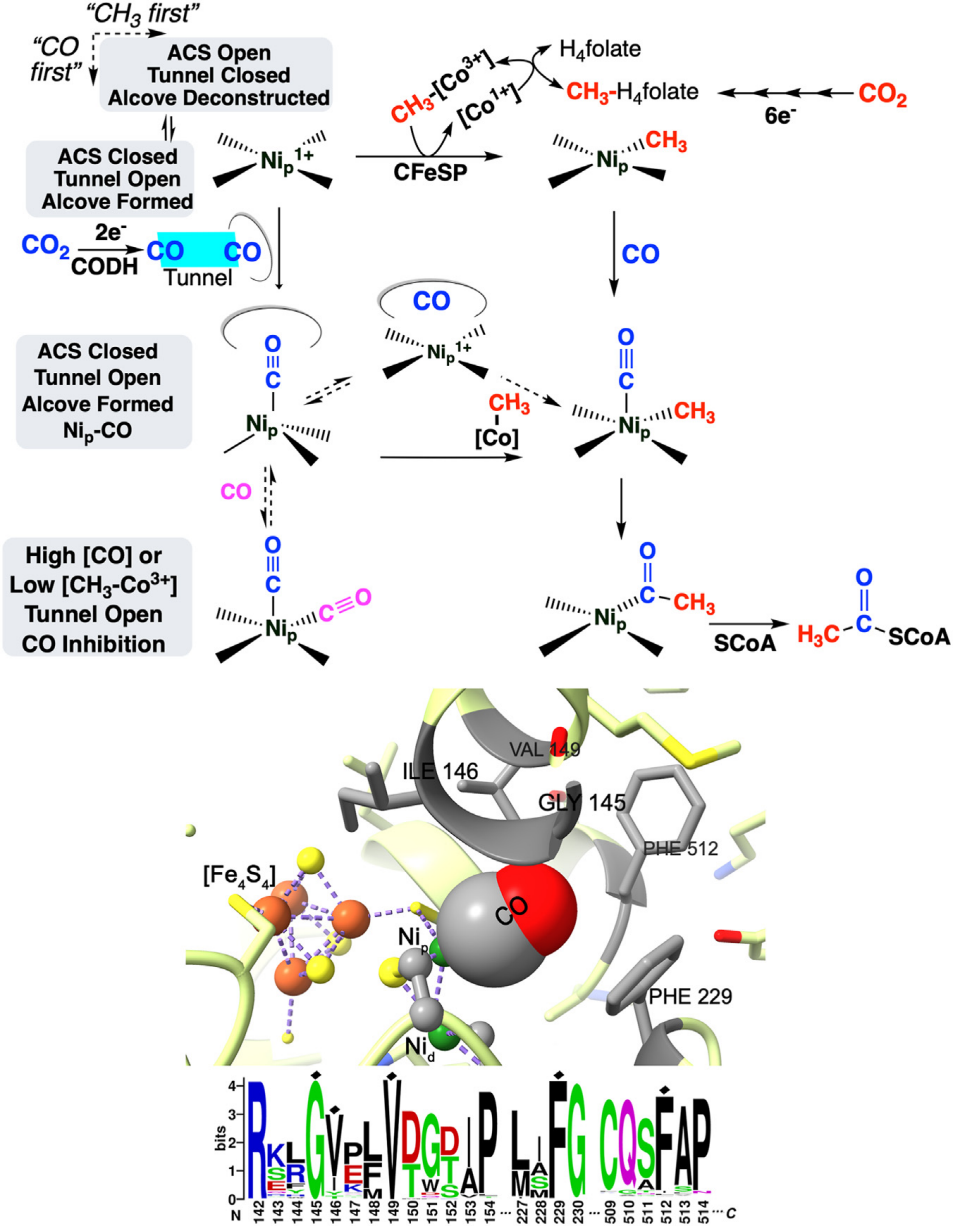
**Alcove requirement for ACS catalysis.** (*Top*), the alcove integrated on one side with Ni_p_ and the A-cluster metallocofactor as the ACS active site and on the other with the tunnel, serving as an extension of the active site. The alcove significantly lowers the K_s_ for CO. The directing actions of the tunnel combined with the concentrating and protective effects of the alcove near Ni_p_, prevents CO loss and inhibition, ensuring ACS can randomly bind either substrate (CO or methyl) at the low CO concentrations required for autotrophic growth. See the text for details. (*Bottom*), conserved sequences containing the five alcove residues. A blast search and multiple sequence alignments were performed of 5000 prokaryotic genomes (excluding archaea) against the *Moorella thermoacetica* ACS (AcsB) sequence (mta_Moth_1202, (55). Sixty amino acids were then loaded into the Weblogo program (56) to visualize the 27 to 28 amino acids with the highest conservation in each of the three Alcove sequence motifs. ACS, acetyl-CoA synthase; AcsB, bacterial ACS.

We propose several potential mechanistic roles for the alcove (Fig. 8). One is to enhance CO binding by increasing the local CO concentration near Ni_p_ of the A-cluster (Fig. 2, *B* and *C*). The inherent CO affinity of the A-cluster (*K*_*D*_ ≥ 340 μM), based on the value for the F229A variant. This is consistent with weak CO binding to a Ni(I)-cyclam (*K*_*D*_ = 30 μM) (33) and a Ni-substituted M121A azurin variant (*K*_*D*_ =1 mM) (34). However, its CO-binding affinity can be tuned at least 2000-fold by site-specific substitutions of a single alcove residue (F229) without affecting other steps in the catalytic mechanism. While anaerobic microbes evolved this mechanism to enhance CO binding by ACS, aerobic organisms positioned distal residues in O_2_-binding proteins, *e.g.* Mb and hemoglobin, to sterically lower their CO affinity, relative to that of the heme metallocofactor, to protect against CO toxicity (35).

Other likely roles of the alcove are to protect ACS from loss of CO and to stabilize the changes in metallocenter ligation (tetrahedral Ni(I)-CO, planar Ni-CH_3_, and planar Ni-acetyl) and overall protein conformation (closed, open, extended, and hyperextended (29)), that it transits during catalysis. Thus, it needs a flexible ligand system that can stabilize both CO- and Me-ACS bound intermediates, including the proposed pentacoordinate methyl-Ni-CO that would undergo CO insertion to generate the Ni-acetyl state (Fig. 8). Having an alcove at the interface of Ni_p_ and the gas tunnel may prevent CO depletion, which would be devastating for autotrophic growth by the WLP, given the significant energy expenditure of acetogens to accomplish the thermodynamically demanding CODH-catalyzed reduction of CO_2_ to CO (E_o_’ = −540 mV). The inability of F209A to grow autotrophically likely results partly from loss of CO from the active site during acetyl-CoA synthesis. ENDOR and EPR studies indicate that F229A binds CO both weakly and haphazardly with CO migrating between the CO and methyl binding sites (16). We envision the alcove grasping CO as it leaves the gas tunnel and approaches Ni_p_ of the A-cluster, as our hands grasp a baseball.

Another role for the alcove highlighted in Figure 8 is to protect ACS from CO inhibition. There has been much discussion about whether methyl and CO bind randomly, or sequentially with methylation occurring first, followed by ordered binding of CoA to the acetyl-Ni species. The random mechanism is supported by pulse-chase experiments in which ^14^CO_2_ incorporation into acetyl-CoA was unaffected by the presence of ^12^CO in solution and ^14^CH_3_ incorporation was unaffected by increasing levels of ^12^CH_3_-Co(III), with strictly ordered binding of CoA (14). Similarly, stopped-flow and freeze quench EPR experiments demonstrate that either methylation or carbonylation to form the methyl-Ni or Ni-CO intermediates at rates faster than the overall steady-state rate of acetyl-CoA synthesis (19, 20). Furthermore, recent XAS and X-ray crystallographic studies have revealed these organometallic Ni(I)-CO, methyl-Ni, and acetyl-Ni complexes (11, 12, 13). On the other hand, evidence supporting an ordered mechanism includes the finding that high CO concentrations can competitively inhibit methylation (23, 36, 37, 38), leading some to propose that the ACS mechanism is strictly ordered with methylation occurring first. Our work resolves these divergent viewpoints and apparent contradictions because the potentially inhibitory CO can escape into the alcove and rebind to Ni_p_ with a nearly barrier-less activation energy of <1 kJ/mol (15). If indeed the organometallic mechanism on Ni_p_ does requires methylation to occur first, when the wrong substrate (CO) binds, the ACS mechanism would still accommodate random substrate binding in which the alcove would provide a temporary harbor for CO, allowing formation of methyl-Ni and then rebinding of CO to form acetyl-Ni.

As shown in Figure 8, this process is exquisitely orchestrated. Prior crystallographic and electron microscopic studies (29) indicating large conformational changes associated with opening and closing of the A-cluster domain of ACS at each catalytic cycle. Based on their location in this domain, the five alcove residues would close like the fingers of a hand around incoming CO as it grasps a ball, holding it near Ni_p_ and enhancing formation of the tetrahedral Ni-CO species. They would open to allow Ni_p_ to bind methyl-Cob(III) (and the CFeSP). Then, to bind CO, they approach in unison like the fingers of a hand as it grasps a ball, holding it near the axial position at Ni_p_ for precise delivery in a *cis* position to the methyl-Ni-CO complex. This sequence of events would be reversed for the “methyl-first” scenario, but in either case, carbonyl insertion would form the planar acetyl-Ni_p_ for reaction with CoA to generate acetyl-CoA.

CO binding is rate limiting in the ACS mechanism and in the WLP; ACS methylation and CoA thiolysis of Ni-acetyl to form acetyl-CoA occur 10- and 100-fold faster (9, 20, 39). Thus, our demonstration that disruption of Ni-CO formation obstructs the WLP suggests that enhancing CO binding (as per the F229W mutation) or delivery through the gas tunnel, could improve the pathway. These strategies are not only important for understanding mechanistic features of this remarkable pathway but suggest opportunities to boost CO_2_ capture and utilization for the benefit of the climate and biotechnology.

Related to CO inhibition, while all alcove residues are highly conserved among bacteria that use the WLP, the homologous CdhC subunit of the methanogenic ACS lacks the N-terminal domain of the bacterial ACS (AcsB). This domain contains four of the five alcove residues and participates in the conformational change that allows interaction with the CFeSP. Mutation of Phe195 (F512 in *M. thermoacetica*), the only alcove residue present in the homologous methanogenic subunit, lowers affinity of AcsB for methyl-Co(III) and enhances CO inhibition of acetyl-CoA synthesis apparently by competing with methylation (23). Thus, either the methanogens do not use an alcove and avoid the striking conformational changes observed in the bacterial ACS (29)or, as others have suggested (40, 41), another subunit of the methanogenic enzyme complex provides these functions. We eagerly await a structure of this five-subunit complex to understand the molecular dynamics and architecture underlying acetyl-CoA synthesis and utilization in methanogens.

In a nutshell, this work reveals how an alcove augments and tunes the inherent gas-binding properties of a metallocofactor, indicates ways to enhance the metabolism of gaseous substrates, and prompts researchers to look for alcoves in other gas-handling proteins that could be considered part of the metalloenzyme active site.

## Experimental procedures

### Generation of monofunctional ACS alcove variants

A pET29 vector (Kan^R^, His6-tagged, *lac*-inducible) harboring the *M*. *thermoacetica acsB* gene containing the ACS subunit of CODH/ACS, known as pET29ACSMT_HT_ was used as a basis for generation of ACS phenylalanine-229 variants. ACS variants F229A and F229W were generated with a QuikChange II site-directed mutagenesis kit from Agilent Technologies using primers (Integrated DNA Technologies) containing the sequences for F229W and F229A, respectively: F229W-*sense* 5′- C CTG CGG GCT GGT ATG ATG TGG GGT -3′ *antisense* 5′- GT AAC GCC ACC CCA CAT CAT ACC AGC -3’; F229A-*sense* 5′- G CGG GCT GGT ATG ATG GCC GGT GGC -3′ *antisense* 5′- GGT AAC GCC ACC GGC CAT CAT ACC A -3’. WT ACS was left unadulterated. The ACS variant plasmids were, respectively, transformed into competent BL21(DE3) *Escherichia coli* cells along with the pBD1282 plasmid (Amp^R^, *ara*-inducible) containing the genes *iscS-iscU-iscA-hscB-hscA-fdx* from *Azotobacter vinlandii* for proper [Fe_4_S_4_] construction (14).

### Growth of *C. autoethanogenum*

Growth of *C. autoethanogenum* was carried out under strictly anaerobic conditions as described earlier using a modified version of ATCC medium 1754 with yeast extract and fructose omitted (42) and a derivative strain (43) of *C. autoethanogenum* type strain DSM10061 obtained from DSMZ (Deutsche Sammlung von Mikroorganismen und Zellkulturen). When noted, 10 g L^−1^ of fructose was added to the medium. For plasmid maintenance, thiamphenicol was added to a concentration of 15 mg L^−1^. Tests for autotrophic and heterotrophic growth were performed in 12-well round-well plates with each well containing 2 ml of growth medium. Wells were inoculated in a glovebox (Inert) with a N_2_ atmosphere, then the plates were placed in custom pressure-rated stainless-steel jars. For each genotype, we tested three biological replicates. The headspace of the jar was replaced with 170 kPa (gauge) of a synthetic CO/CO_2_/H_2_/N_2_ (50%/30%/10%/10%) gas blend (Airgas) for autotrophic growth conditions and left with ambient pressure of N_2_ (Airgas) for heterotrophic growth on fructose. Jars were shaken at 90 rpm with a 2.5 cm throw and incubated at 37 °C for growth.

### Genetic modification of *C. autoethanogenum*

Homologous recombination, with counter selection, facilitated deletion of *acsB* in *C. autoethanogenum* with target-specific modifications from the method previously described (44). Namely, we amplified homology arms from genomic DNA with the oligonucleotides listed in Table S2 and used TCACCAAATGTCATTCCAAG as the recognition sequence for the guide RNA. The vector was assembled with the GeneArt Seamless Cloning kit (Thermo Fisher Scientific). We included fructose in the medium during deletion of *acsB* since we anticipated the essentiality of the gene for autotrophic growth. For expression of *acsB*, we amplified the gene from genomic DNA and cloned it into pMTL83151-P_acsA_ (27) using oligonucleotides listed in Table S2. We created the F209A substitution using the kinase, ligase, and DpnI (KLD enzyme mix) kit (New England Biolabs) with oligonucleotides listed in Table S2.

### Expression and purification of monofunctional ACS variants in competent *E. coli* cells

Growth of *E. coli* cells harboring the ACS variant plasmids was conducted as previously described (14). Lysis and purification of ACS and variants were performed as previously described with protein concentrations verified by the Rose Bengal assay (45). All buffers and reagents were prepared under strict anaerobic conditions (≤2.5 ppm O_2_), and all glassware used in purification was acid washed for no less than 1 h to remove any metal contaminants.

Metal reconstitution of pure ACS was performed as described previously (46). ACS metal content post reconstitution is quantified by inductively coupled plasma optical emission spectroscopy through the Center for Applied Isotope Studies at the University of Georgia (Athens, GA) in comparison to the protein concentration verification by the Rose Bengal assay (45).

### EPR analysis of natural abundance and isotopically labeled ACS-CO variants

Continuous-wave X-band EPR data were collected on a Bruker EMX equipped using a variable-temperature ColdEdge liquid helium condensing unit with a MercuryiTC cryostat by Oxford Instruments. All spectra were collected using the WinEPR software package (https://www.bruker.com/en/products-and-solutions/mr/epr-instruments/epr-software/winepr.html). EPR samples were prepared as previously described (21) by anaerobic mixing of ACS in 50 mM potassium phosphate pH 7.5 with 2 mM sodium dithionite, transferred to an EPR tube capped with a rubber septum, and purged with 100% carbon monoxide gas (ultra-high purity; Cryogenic Gases) for 25 min before being flash frozen in liquid nitrogen. Protein concentration was assessed in parallel by the Rose Bengal assay (45). Isotopically labeled ACS variants were treated as previous CO samples, but purge time was reduced to 10 min to minimize wasted ^13^CO gas. All samples were stored at −80 ˚C prior to spectrum acquisition. Natural abundance (NA) WT and F229W EPR spectra were collected at 40 K with a power of 20.8 mW, a modulation frequency of 100 kHz, a modulation amplitude of 10 G, gain of 2000, conversion time of 81.92 ms, and a time constant of 40.96 ms. NA F229A EPR spectrum was collected at 100 K with a power of 20.8 mW, a modulation frequency of 100 kHz, a modulation amplitude of 10 G, gain of 4480, conversion time of 40.96 ms, and a time constant of 20.48 ms. ^13^CO WT EPR spectrum was collected at 100 K with a power of 2.08 mW, with a modulation frequency of 100 kHz, a modulation amplitude of 10 G, gain of 4480, conversion time of 163.84 ms, and a time constant of 40.96 ms. The ^13^CO F229W and F229A spectra were collected at 100 K with a power of 20.8 mW, modulation frequency of 100 kHz, a modulation amplitude of 10 G, gain of 4480, conversion time of 40.96 ms, and a time constant of 20.48 ms. Analysis of EPR spectra was conducted using the WinEPR software package, and the resultant ACS spectra were quantitatively measured by comparison to a 1 mM Cu(II) perchlorate standard using the equation from Fee, 1978 (47).

For spin quantification by EPR, an initial 10 mM Cu(II) perchlorate standard stock was prepared for spin quantitation by mixing together 12.24 g sodium perchlorate (Sigma-Aldrich), 0.125 g Cu(II) sulfate pentahydrate (99.999% purity; Sigma-Aldrich), and 42 μl saturated (12 M) HCl with ultrapure water to a final volume of 50 ml. Subsequently, 10 mM Cu(II) perchlorate stock was diluted by 10 to 1 mM standard with high purity water, where a 200 μl aliquot is transferred to an EPR tube and frozen with liquid N_2_.

### CO titration of ACS WT and variants monitored by EPR

The headspace of the protein samples in 50 mM Tris, pH 7.6 with 20% glycerol and 5 mM dithionite (concentration measured by Rose-Bengal assay) were exchanged for varied lengths of time (from 15 s to 10 min) with a 5% CO/N_2_ gas mix and 100% CO gas to achieve wide-ranging CO concentrations. The final CO concentrations were measured by CO binding to equine Mb at 424 nm, prior to flash-freezing. All samples were stored at −80 °C prior to acquisition. The continuous-wave X-band EPR data were acquired on a Bruker EMX equipped with a liquid nitrogen cryostat. All spectra were collected using WinEPR Acquisition software. WT spectra were collected at 100K with a single scan at a power of 0.644 mW, a modulation frequency of 100 kHz, a modulation amplitude of 5 G, gain of 1 x 10^5^, conversion time of 163.84 ms, and a time constant of 81.92 ms. F229W spectra were collected at 100K with a single scan at a power of 0.644 mW, a modulation frequency of 100 kHz, a modulation amplitude of 5 G, gain of 1 x 10^5^, conversion time of 81.92 ms, and a time constant of 81.92 ms. F229A spectra were collected at 100K with four scans at a power of 0.644 mW, a modulation frequency of 100 kHz, a modulation amplitude of 5 G, gain of 1 x 10^5^, conversion time of 81.92 ms, and a time constant of 81.82 ms. Analysis of EPR spectra was conducted using WinEPR Processing software, and the resultant ACS spectra were quantitatively measured by comparison to a 1 mM Cu(II) perchlorate standard using the equation from Fee, 1978 (47). The quantified spin percentages were plotted against the concentration of CO for each sample. Since the enzyme concentration is greater than the calculated KD for each of the variants, the free-ligand assumption for a simple binding isotherm does not apply; the data were fitted to the quadratic tight-binding equation (Equation 1) to reveal average K_D_ values (with 95% *CIs*) of 12.8 μM (7.3–20.2 μM), <1 PM (<1 pM-8 μM), and 340 μM (140–940 μM), respectively, for WT, F229W, and F229A.

(1) $$y=\frac{\left(E_{T}+x+K_{d}\right)-\;\sqrt{{\left(E_{T}+x+K_{d}\right)}^{2}-4*E_{T}*x}}{2}$$

where x = [CO] (uM)

#### CO competition assays with equine Mb

Because the direct CO binding experiments (Fig. 4*A*) returned such wide CIs of the K_D_(CO) for F229W, scaling orders of magnitude, we used anaerobic equilibrium CO competition experiments between F229W and dithionite-reduced (Fe^2+^) equine Mb, which exhibits a K_D_(CO) of 22 nM (26). The titrations were measured by UV-visible and EPR spectroscopy.

##### *UV-visible measurement of Mb-CO*

The competition experiment was performed in the anaerobic chamber (<1 ppm O_2_) using stock solutions of 800 μM equine Fe^2+^-Mb (Sigma-Aldrich, EC 309-705-0) reduced with 500 mM dithionite; 77 μM active A-cluster of F229W (from 336 μM F229W, measured by EPR as the Ni(I)-CO complex with saturating CO); and CO (1140 μM). All stock solutions were prepared in 50 mM Tris–HCl, pH 7.6. For the triplicate Mb-CO control samples, Fe^2+^-Mb (7.5 μl, 4 μM, final) was added anaerobically *via* a 10 μl Hamilton syringe to 1187 μl of buffer (50 mM Tris–HCl, pH 7.6) and the cuvette was sealed with a rubber sleeve stopper. The initial spectrum of reduced Fe^2+^-Mb was recorded; then CO (5.3 μl, 4.0 μM, final) was added to make Mb-CO. The difference spectrum (*i.e.*, the spectrum of reduced Fe^2+^-Mb-CO minus from Fe^2+^-Mb)_represents the maximum Mb-CO and was used to calculate the fraction of Mb-CO during the equilibrium experiment with ACS. For the quintuplicate competition experiments, F229W (78 μl, 4 μM active A-cluster, final) was added to 1402 μl buffer in a 1 cm path-length cuvette, sealed with a rubber sleeve stopper; then CO (5.3 μl, 4 μM) was added and equilibrated for 5 min before recording an initial spectrum. Then Mb (7.5 μl, 4 μM) was added and incubated for 30 min before recording the equilibrium spectrum. Some samples were remeasured after 24 h, and we saw no further spectral changes. All spectra were adjusted for dilution to a final volume of 1500 μl. The initial CO-F229W spectrum was subtracted from the equilibrium spectrum to isolate the absorbance from Mb during competition with F229W. The 4 μM reduced Mb spectrum was subtracted from the equilibrium competition Mb spectrum to quantify the amount of Mb-CO at equilibrium. The fraction of Mb-CO (f_myo_) during competition with F229W was compared to the Mb-CO controls and Equation 2 was used to calculate the apparent K_D_ for CO binding to F229W-ACS, where the K_D_,_*myo*_ (K_D_(CO) for equine Mb-CO) is 22 nM (26), [CO] is 4 μM, and f_myo_ is the fraction of Mb bound by CO. Based on the five UV-visible experiments, the K_D_(CO) for F229W was 160 ± 30 nM (95% CI).

(2) $$K_{D,F229W}=\frac{K_{D,myo}*\left[CO\right]}{f_{myo}-K_{D,myo}}$$

##### EPR measurement of F229W-CO

The amount of F229W-CO was determined in triplicate equilibrium binding EPR experiments in which 200 μl samples were transferred from the cuvette to a quartz capillary and quickly frozen (30 s). The EPR samples (four scans) were run at 15 K, 0.4 mW power, 10 G modulation amplitude, 9.372 GHz frequency. Two of the three samples had a measurable A-cluster signal which was quantified against a 1 mM Cu perchlorate standard. For the third sample, the anaerobic syringe became clogged, and we reverted to a micropipette, which may have led to CO loss during the transfer, causing the signal to be too low to accurately quantify above the noise. For the other two samples, the μM spin was used to calculate f_myo_, and Equation 1 was used to calculate the apparent K_D_ for CO binding to F229W. The relevant parameters for sample 4 were 0.86 μM spin giving f_myo_ = 0.79 and a 115 nM K_D_ for F229W-CO (the UV-visible had 2.5 μM CO and an f_myo_ = 0.63; for sample 5 were spin = 2.18 μM giving f_myo_ = 0.46 leading to a 203 nM K_D_ for F229W-CO (the UV-visible had 2.3 μM CO and an f_myo_ = 0.57). Thus, based on EPR measurements, the average K_D_ value was 160 ± 90 nM (95% CI). Because of the signal/noise of the tiny EPR signals, there was a larger 95% CI; furthermore, there were two EPR samples compared to five UV-visible experiments. Regardless, the f_myo_ between the EPR and UV-visible results is within the random error associated with the EPR measurements. Finally, to compare the equilibrium binding results to those obtained from the direct CO titrations, the quadratic fit of the direct CO titration data using a K_D_ of 160 nM (Fig. 4*A*, dotted line) returned a similar R^2^ as that for the unconstrained fit (K_D_ of 1 PM, solid line) with an unreasonably high CI.

Thus, the three K_D_(CO) measurements (from the direct CO binding and CO competition experiments) are consistent with a K_D_(CO) for the F229W-CO complex between 130 to 190 nM at a 95% confidence level, which demonstrates a CO affinity 80-fold higher than wt F229 and 2125-fold higher than F229A.

### XAS measurements

XAS spectra were measured at the Stanford Synchrotron Radiation Lightsource on the focused 16-pole, 2 T wiggler side-station beam line 9-3 under standard ring conditions of 3 GeV and ∼500 mA. A Si(220) double crystal monochromator was used for energy selection. A Rh-coated harmonic rejection mirror was used to reject components of higher harmonics. During data collection, the samples were maintained at a constant temperature of ∼10 K using an Oxford Instruments CF 1208 liquid helium cryostat. A 100-element Ge monolith fluorescence detector from Canberra Industries was used for fluorescence data measurement.

#### Fe K-edge measurements

Fe K-edge XAS data were measured on the dithionite reduced (ACS_red_) and dithionite reduced followed by CO exposure (ACS_red_-CO) in the fluorescence mode by using soller-slits equipped with Mn-filter in front of the Ge detector and using an Fe-foil for energy calibration. The first inflection point of the foil spectrum was fixed at 7111.2 eV. The samples were monitored for photoreduction and showed no signs of change during data collection. Fe K-edge EXAFS data were measured up to *k* = 15 Å^−1^. The data presented here are 8-scan (WT) and 4-scan (variants) average spectra, collected over four fresh-sample positions for each of the samples.

#### Ni K-edge measurements

Ni K-edge XAS data were measured in fluorescence mode by using soller-slits equipped with Co-filter in front of the Ge detector and using a Ni-foil for energy calibration. The first inflection point of the foil spectrum was fixed at 8331.6 eV. The samples were monitored for photoreduction and no sign of photodamage or Ni-CO bond-cleavage was observed during the course of data collection. Ni K-edge EXAFS data were measured up to *k* = 18 Å^−1^. The data presented here are 12-scan (WT) and 9-scan (variants) average spectra, collected over four fresh-sample positions for each of the samples.

#### XAS data reduction

Data presented in this study were processed by fitting a second-order polynomial to the preedge region and subtracting this from the entire spectrum as background in PySpline (48). A four-region spline of orders 2, 3, 3, and 3 was used for both the Ni and Fe datasets to model the smoothly decaying postedge region. Theoretical Ni K-edge EXAFS signals *χ*(*k*) were calculated by using *FEFF* (macintosh version 7) (49, 50) on the active site structure derived from the crystal structure of ACS (pdb = 1RU3). Structural models were generated for the different putative forms of Ni_p_ and Ni_d_ sites. The molecule editing software Avogadro (51) was used to modify the structural parameters obtained from the crystal structure. Theoretical models were fit to the data using EXAFSPAK (52). The structural parameters varied during the fitting process were the bond distance (R) and bond-variance *σ*^2^, which is related to the Debye-Waller factor resulting from thermal motion, and static disorder of the absorbing and scattering atoms. The nonstructural parameter ΔE_0_ (the energy at which *k* = 0) was also allowed to vary but was restricted to a common value for every component in a given fit. Coordination numbers were systematically varied in the course of the fit but were fixed within a given fit.

### XAS sample preparation

XAS samples of the F229W and F229A variants of activated ACS were prepared as previously described (11): two protein states were prepared for each; ACS_red_ and ACS_red_-CO. ACS_red_ was the protein reduced with 2 mM sodium dithionite, while ACS_red_-CO was similarly reduced and then purged with pure CO for 10 min in a sealed and crimped vial. Final variant concentration was 1 mM after addition of 30% glycerol as a glassing agent in 50 mM potassium phosphate, pH 7.5, and protein concentration was verified by the Rose Bengal assay (45). Variant samples were quickly placed in XAS sample cells, removed from the anaerobic chamber, flash frozen in liquid nitrogen, and shipped overnight to the Stanford Synchrotron Radiation Lightsource for data acquisition and analysis.

### FTIR sample preparation

FTIR ACS variant samples were prepared by buffer exchange of variants into 50 mM potassium phosphate in D_2_O, pH 7.7, then concentration of the variants to an expected 1 mM using ultracentrifugal filters with a 30 kDa cutoff membrane, spun in a tabletop centrifuge at 10,000 rpm for 4 min. Concentrated ACS samples were purged with pure CO gas for 10 min in stoppered and crimped 2 ml vials, where the final ACS concentration was assessed by the Rose Bengal assay (45). After CO purging, the samples were transported in airtight containers to a collaborator’s anaerobic chamber, where the samples were transferred to a CaF_2_ FTIR sample window. Once in the FTIR window, the sample was removed from the chamber and the IR spectrum taken immediately (JASCO FT/IR-4600) compared to a D_2_O buffer blank.

### Methylation of WT and variant ACS

Methylation of ACS variants was performed in darkness with red light by Stopped-Flow UV-visible spectrophotometry (Applied Photophysics SX20; single-wavelength monochromator), using MeCbi as a methyl donor, and titanium(III) citrate (Ti(III)) citrate as a reducing agent. A reaction solution consisting of 10 μM ACS and 30 μM Ti(III) citrate was prepared in 50 mM potassium phosphate, pH 7.5. Reaction solutions containing ACS were blanked, and the experiment began with addition of 10 μM MeCbi. Resultant spectral changes associated with Cbi ligand changes and redox shift from 3+ to 1+ state and were quantified using the change in extinction coefficient at 390 nm (Δε_390_ = 17 mM^−1^ cm^−1^), *i.e.*, for methyl-Cob(III)inamide, ε_390,MeCbi_ = 8 mM^−1^ cm^−1^ and for Cob(I)inamide ε_390,Co(I)bi_ = 25 mM^−1^ cm^−1^. The spectral changes were also quantified at 470 nm (ε_470,MeCbi_ = 11 mM^−1^ cm^−1^; ε_470,Co(I)bi_ = 3 mM^−1^ cm^−1^; Δε_470_ = 8 mM^−1^ cm^−1^).

Calculation of percentages associated with methylation of ACS was performed using the following equation:

$$Conversion_{390}\;\%=\left(\frac{\left(\left(\frac{Abs@390-Abs_{0}@390}{\varepsilon_{Co\left(I\right)}-\varepsilon_{MeCo\left(III\right)}}\right)*1000\frac{\mu M}{mM}\right)}{{\left[MeCo\left(III\right)binamide\right]}_{0}}\right)*100=\left(\frac{\left(\left(\frac{Abs@390-Abs_{0}@390}{\left(25-8\right)mM^{-1}}\right)*1000\frac{\mu M}{mM}\right)}{10\;\mu M\;MeCbi}\right)*100$$

### Preparation of MeCbi and Ti(III) citrate reductant

MeCbi was prepared as described (53) with an anaerobic 40% methyl iodide/60% methanol solution prepared on ice by blowing high purity nitrogen over the mixture for 5 min. Once the 40% methyl iodide/methanol solution is prepared, the MeCbi is made anaerobically by mixing 1x (mol/mol) dicyano-Cbi (≥93%; Sigma-Aldrich) with 6x (mol/mol) Ti(III) citrate in a brown glass vial. The vial is then stoppered with rubber septum, and 240x (mol/mol) of the previously prepared 40% methyl iodide/methanol solution is added and left to react at room temperature for ≥ 2 h. After reacting, the solution is then purified *via* size-exclusion with 50 mM potassium phosphate buffer, pH 7.5 under green light by an anaerobic Bio-Gel P2 (Bio-Rad; 100–1800 MW cutoff) and collected in 0.5 ml aliquots and checked for purity *via* UV-visible spectroscopy using ε_390_ = 8 mM^−1^ cm^−1^ and ε_470_ = 10.8 mM^−1^ cm^−1^. Pure and concentrated MeCbi aliquots are pooled, quantified, and stored at −20 ˚C.

Ti(III) citrate (83 mM stock) reductant was prepared anaerobically as previously described (54). Ti(III) citrate solutions were stored anaerobically at room temperature away from light in a brown glass vial.

## Data availability

All data are presented in the Main Manuscript or the Supporting Information. Materials are available upon reasonable request and under a material transfer agreement, but strains may require a license.

## Supporting information

This article contains supporting information (29).

## Conflict of interest

A. P. M., R. N., S. D. S, M. K. are employees of LanzaTech that has commercial interest in gas fermentation. All other authors declare no conflict of interest with the contents of this article.
