# Supplementary material for: An alcove at the acetyl-CoA synthase nickel active site is required for productive substrate CO binding and anaerobic carbon fixation
Source: J Biol Chem. 2024 Jun 27;300(8):107503. doi: 10.1016/j.jbc.2024.107503 (PMC11321310; doi:10.1016/j.jbc.2024.107503)
Supplement: Supporting Information [file mmc1.pdf]

Supporting Information

An Alcove at the Acetyl-CoA Synthase Nickel Active Site Is Required for Anaerobic CO_2 Fixation

Authors: Seth Wiley[1,2], Claire Griffith[1], Peter Eckert[3], Alexander P. Mueller[4], Robert Nogle[4], Séan D. Simpson[4], Michael Köpke[4], Mehmet Can[1,5], Ritimukta Sarangi[6], Kevin Kubarych[3], Stephen W. Ragsdale[1]*

Affiliations:

[1] Department of Biological Chemistry, University of Michigan, Ann Arbor, MI 48109-0606.

[2] Current Address: Biosciences Center, National Renewable Energy Laboratory, Golden, CO 80401.

[3] Department of Chemistry, University of Michigan, Ann Arbor, MI 48109

[4] LanzaTech Inc., 8045 Lamon Ave, Skokie, IL, USA 60077

[5] Current Address: Department of Biochemistry, Faculty of Pharmacy, Ankara Medipol University, Ankara, Turkey 06050

[6] Stanford Synchrotron Radiation Lightsource, SLAC National Accelerator Laboratory, Menlo Park, CA 94025.

*Correspondence to: Stephen W. Ragsdale (sragsdal@med.umich.edu)

This PDF file includes:

Supplementary Text
Figs. S1 to S6
Table S1 and S2

Supplementary Text

Calculation of Percentages Associated with Methylation of ACS

$$Conversion_{390}\ \% = \left(\frac{\left(\left(\frac{Abs@390 - Abs_0@390}{\varepsilon_{Co(I)} - \varepsilon_{MeCo(III)}} \right) * 1000 \frac{\mu M}{mM} \right)}{[\boldsymbol{MeCo(III)binamide}]_{\mathbf{0}}} \right) * 100$$

$$= \left(\frac{\left(\left(\frac{Abs@390 - Abs_0@390}{(25-8)mM^{-1}} \right) * 1000 \frac{\mu M}{mM} \right)}{\mathbf{10}\ \mu \boldsymbol{M}\ \boldsymbol{MeCbi}} \right) * 100$$

References:

29. S. E. Cohen *et al.*, Negative-Stain Electron Microscopy Reveals Dramatic Structural Rearrangements in Ni-Fe-S-Dependent Carbon Monoxide Dehydrogenase/Acetyl-CoA Synthase. *Structure* **29**, 43-49 e43 (2021).

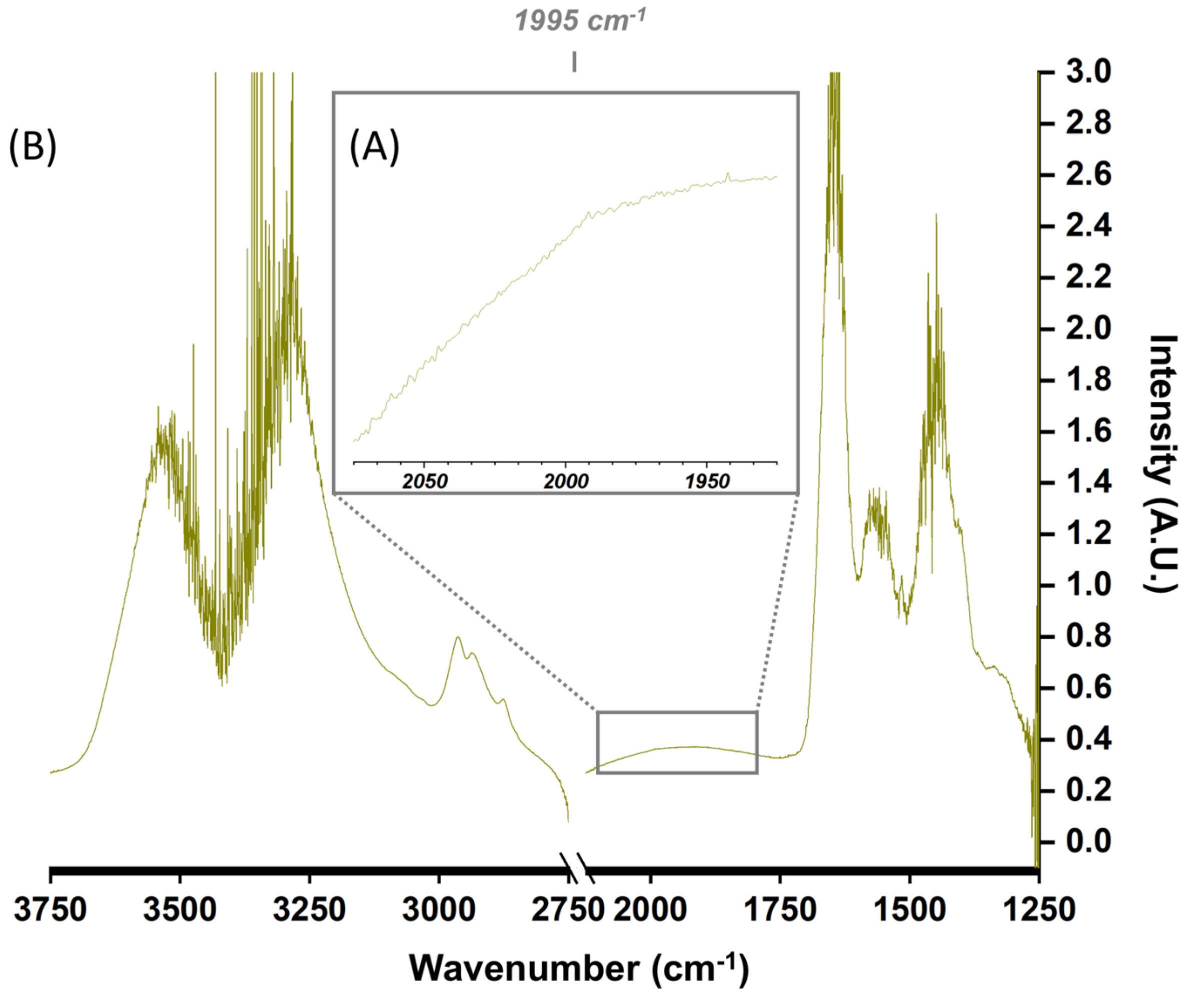

Fig. S1. FTIR spectrum of CO-reacted F229A at the higher concentration of 2.3 mM. (**A**) The region of the Ni-CO stretching frequency, showing no obvious characteristic Ni-CO at 1995 cm^{-1}. (**B**) The full IR spectrum of the 2.28 mM F229A. Despite the relatively high concentration, there was no characteristic Ni-CO peak at 1995 cm^{-1} observed for the F229A variant.

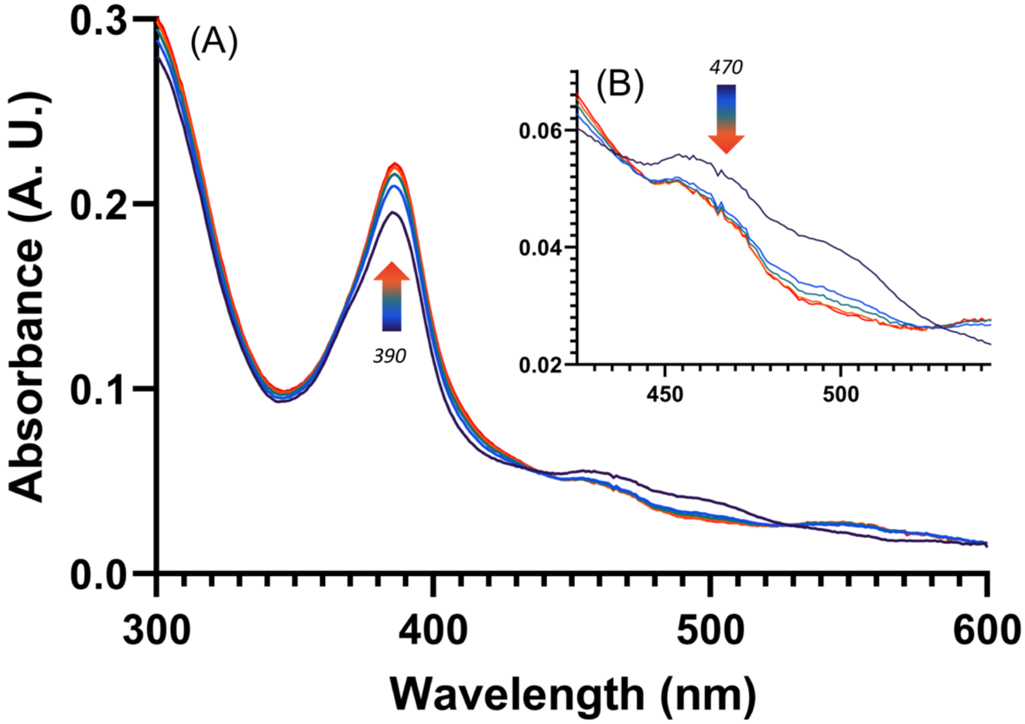

Fig. S2. Monochromatic scan of Stopped-Flow data seen in **Fig. 2B**, scanned over the course of 10 minutes. (**A**) Spectrum of the expected Cob(I)inamide species after ACS is methylated, showing a characteristic increase at 390 nm. (**B**) Inset of the expected decrease at 470 nm showing the decay of methyl-Cob(III)inamide, as well as two isosbestic points at 438 nm and 528 nm observed during conversion of methyl-Cob(III)inamide to Cob(I)inamide.

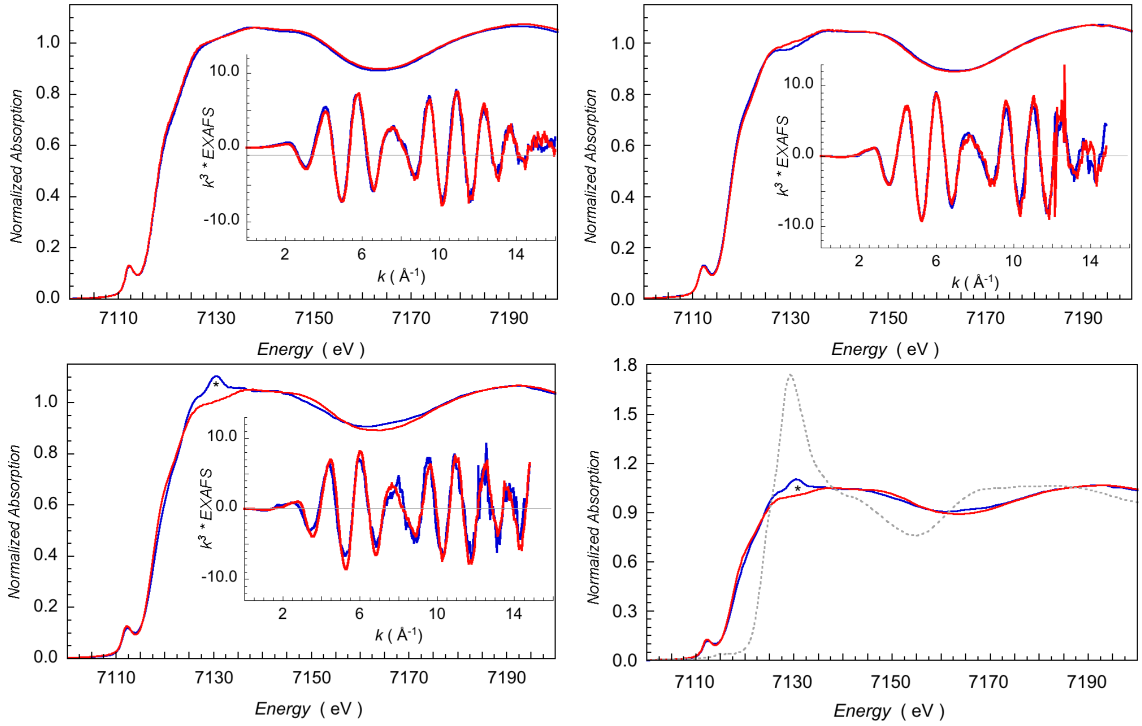

Fig. S3. Fe K-edge XAS data showing no change at the Fe-edge or EXAFS (inset) for the ACS WT (top left), F229A (top right) and F229W (bottom left) upon CO binding. Reduced (blue), Ni-CO (red). The region marked with a * in the F229W reduced form indicates a small signal from extraneous Fe(II) (~18%) present in the cryostat. (Bottom right) The spectra for F229W reduced (blue) and CO treated (red) plotted with a Fe(II) standard to confirm this. Despite this, the EXAFS data are only slightly perturbed in the reduced F229W dataset (bottom left, inset) because the very strong signal from the Fe-S and Fe-Fe of the $[4Fe\text{-}4S]^{2+}$ cluster drown the signal from the Fe(II) contaminant in the EXAFS region.

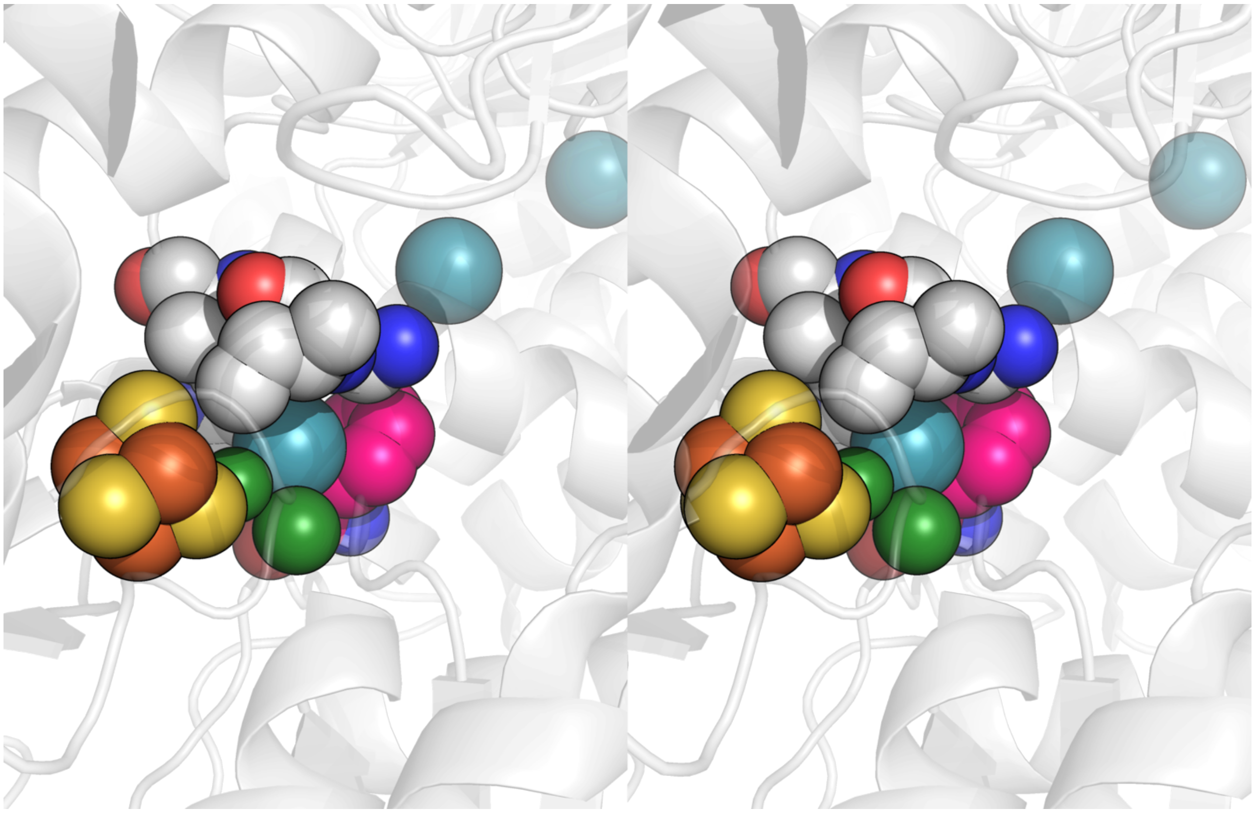

Fig. S4. Stereoscopic view of **Fig. 5a**. PDB 2Z8Y.

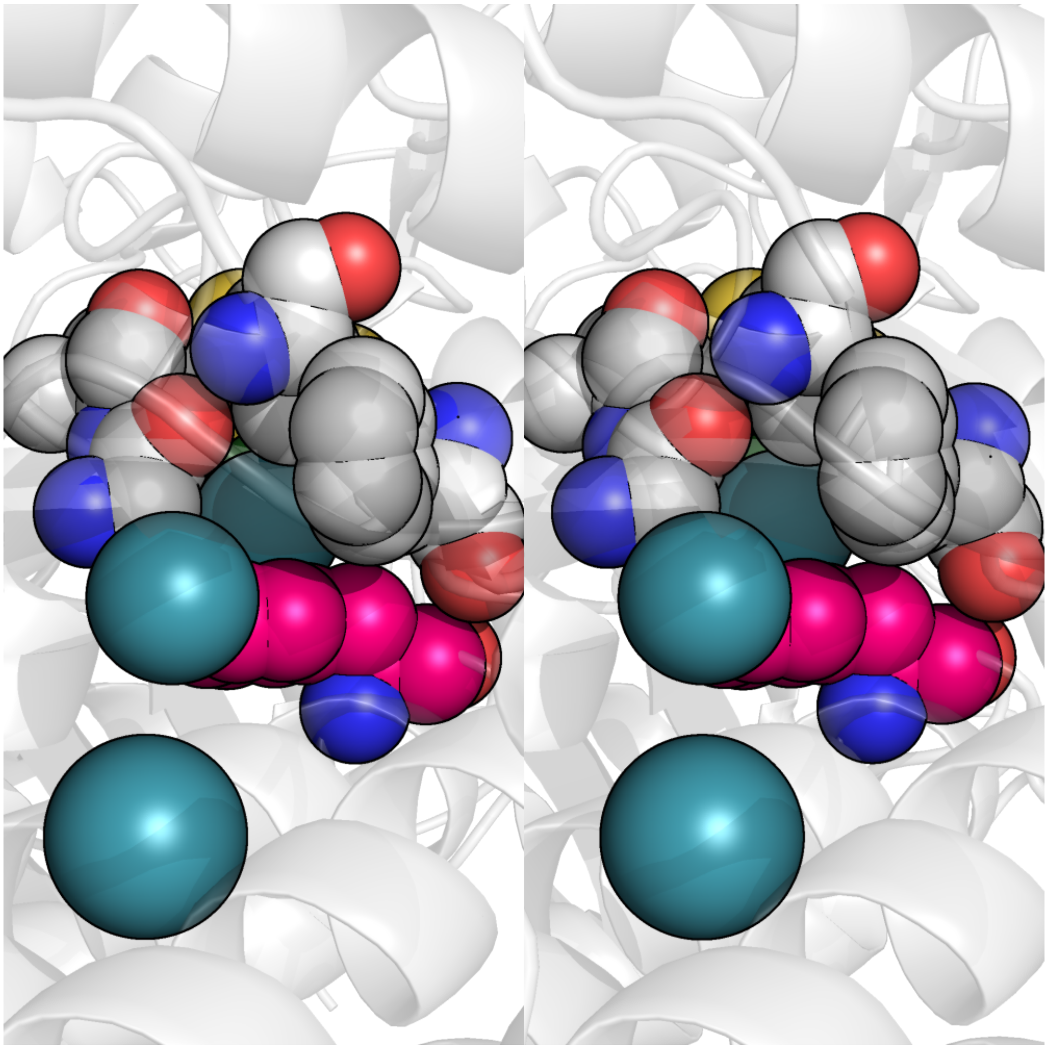

Fig. S5. Stereoscopic view of Figure **Fig. 5(B)**. PDB 2Z8Y

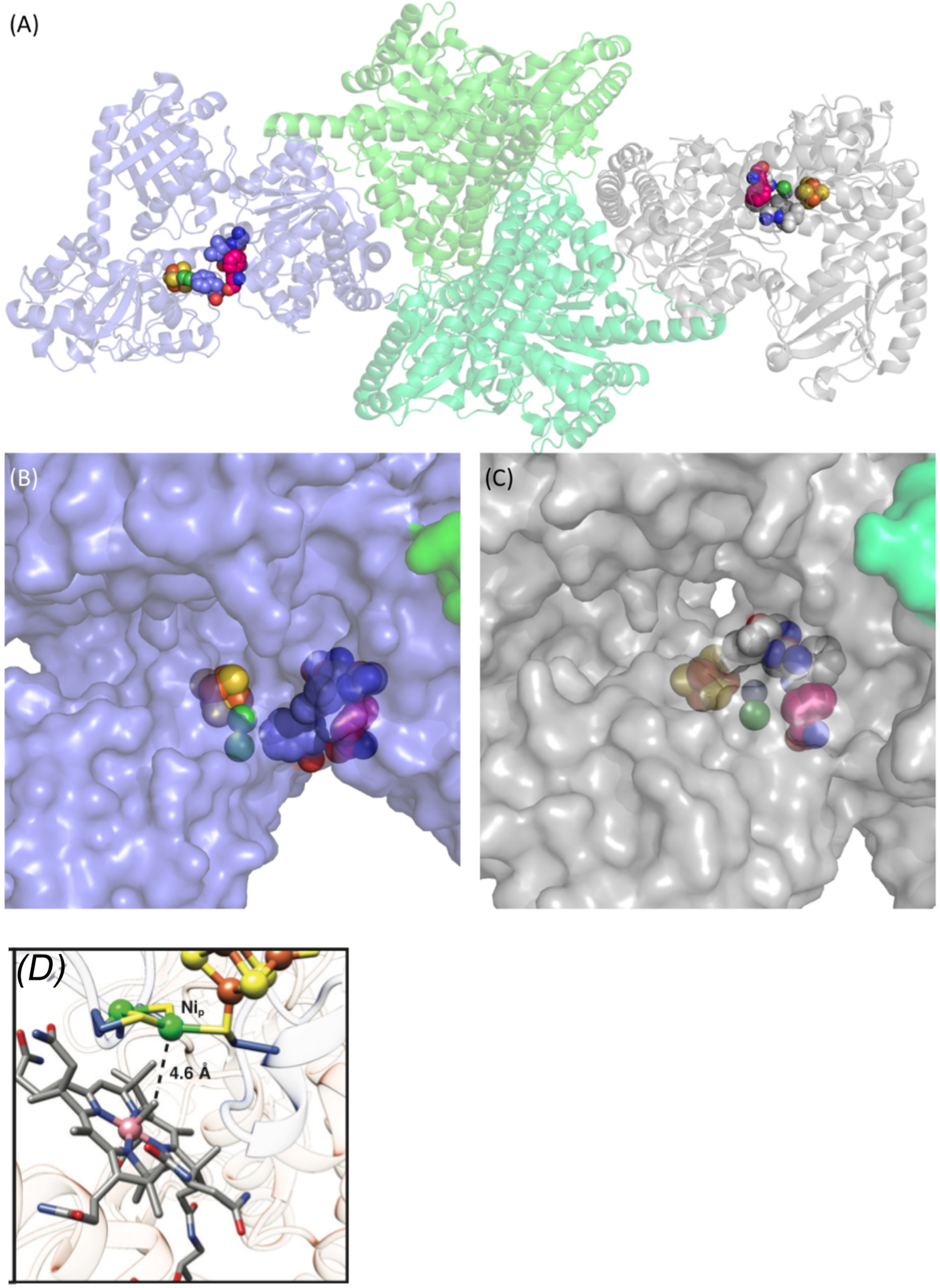

Fig. S6. A-cluster changes associated with Open and Closed ACS. (**A**) Global view of the CODH/ACS heterotetramer, with both open (blue) and closed (grey) conformations of ACS, and

the residues mentioned in this work modeled as spheres (CODH in shades of green). CODH/ACS crystal structure is different than the Xenon structure referenced previously. (**B**) Close-up surface model of the open (blue) conformation showing the exposed A-cluster primed for methylation, with F512 completely shifted from the closed conformation. (**C**) Close-up surface model of the closed (grey) conformation showing the open CO tunnel as seen in **Fig. 8**, **Fig. S4**, and **Fig. S5**. PDB 1OAO. (D) Close-up of the ACS A-cluster and CFeSP CH_3-Co(III)binamide. CFeSP backbone in salmon and CH_3-Co(III) C, N, O, P and Co colored in gray, blue, red, orange, and pink, respectively. Methyl transfer distance from Co of Cba to Nip of the A-cluster is indicated by the dashed line. From Fig. 5 (also see Video S3) of ref 29.

Table S1. Variant Metal Content Determined by ICP-OES.

ACS Variant	Iron per ACS	Nickel per ACS
Expected	*4.0*	*2.0*
Wildtype	3.3 ± 0.4	1.8 ± 0.4
F229W	3.0 ± 0.7	1.7 ± 0.4
F229A*	3.8 ± 0.3	2.4 ± 0.2

**Concentration methods were improved between purifications. Variants were initially concentrated using centrifugal concentrators but were changed from centrifugation to pressure concentration. Pressure concentration appeared to increase metal retention in ACS protein.*

Table S2. Oligonucleotides used for *C. autoethanogenum* genetic modification

Oligo name	Sequence
Gene deletion (binding sequences in bold)	
Left homology arm F	CTTCTTATTTTTATGGCGCGCC**ATTTTCCACAGGACCCACTTCC**
Left homology arm R	AAGATAACTAATG**CTTGCATCTATGACAGGTGGTGG**
Right homology arm F	GATGCAAG**CATTAGTTATCTTTTTCCTGTAGCTGC**
Right homology arm R	TTGTTCAAAAAAATAATGGC**TGAACATGGATAAATTTATGATCATAGGCG**
acsB cloning (binding sequences in bold)	
acsB F	GGAGGGAATTATTCATATGAATTTATTTCAAACTGTATTCACTGG
acsB R	CAGTCACGACGCTACATTATTGGATCCATCTTTAATGCAG
Vector F	CCAATAATGTAGCGTCGTGACTGGGAAAACC
Vector R	GAAATAAATTCATATGAATAATTCCCTCCTTAAAGAAATAACTTC
acsB mutagenesis (altered codon in bold)	
F209A F	GGAGGAATTAAGGGTGGTCAG
F209A R	**CGC**TATAAGTGCAGCTCTTATAGC
